# Assessment of Genetic Diversity in Quinoa Landraces Cultivated in the Ecuadorian Highlands Since the Early 1980s

**DOI:** 10.3390/plants14050635

**Published:** 2025-02-20

**Authors:** Hipatia Delgado, Juan Pedro Martín

**Affiliations:** Departamento de Biotecnología-Biología Vegetal, Escuela Técnica Superior de Ingeniería Agronómica, Alimentaria y de Biosistemas, Universidad Politécnica de Madrid, Avda. Puerta de Hierro 2-4, 28040 Madrid, Spain; angelica.delgado.pilla@alumnos.upm.es

**Keywords:** *Chenopodium quinoa*, genetic structure, genotypic variability, native quinoa, SSR markers

## Abstract

Quinoa (*Chenopodium quinoa* Willd.) landraces have been cultivated ancestrally in the Andean highlands of Ecuador, where they have had great social and nutritional importance for the native population. Currently, there is scarce information on its genetic diversity and conservation status, and none on the changes that may have occurred in recent decades. In this study, we assessed the genetic diversity of 268 accessions (1340 samples; five per accession) of quinoa landraces collected at two different times (1978–1988 and 2014–2015) in three representative Ecuadorian Andean provinces for this crop (Imbabura, Cotopaxi and Chimborazo) using eight simple sequence repeat (SSR) markers. A total of 124 alleles were found, with a range of 11–24 per locus (mean = 15.5). Averages of expected heterozygosity (*H_e_*) and Shannon information index (I) were 0.763 and 1.821, respectively. The most informative loci were 3_QAAT050 and 7_QAAT100, with discrimination power (D) values above 92%. Moreover, 1055 different genotypes were found, of which 939 were unique genotypes. This high level of genetic diversity could be explained by the intensive exchange of seeds between farmers in the Andean region. In addition, no significant differences were found in the main genetic diversity parameters between collections and/or provinces. If there is no significant quantitative loss of genetic diversity over the last four decades, this would indicate that indigenous farming communities of this Andean region are preserving their ancestral quinoa germplasm reasonably well. Furthermore, two genetic groups were found with a genetic distance of 0.337. Samples in these groups appear to be related to their provincial origin. This may be due to the different ways in which quinoa landraces are managed and conserved in the different Andean provinces. The results obtained may be very useful for the suitable management and conservation of this ancestral plant genetic resource, both on farm by indigenous farming communities and ex situ by the Germplasm Bank of the Ecuadorian National Institute for Agricultural Research (INIAP).

## 1. Introduction

Quinoa (*Chenopodium quinoa* Willd.) is an allotetraploid species (2n = 4x = 36), with its center of origin located in the Andes of Bolivia and Peru [1,2]. Archaeological evidence suggests that the domestication of quinoa began more than 7000 years ago in the Andean Altiplano by pre-Columbian cultures and subsequently spread throughout western South America [3,4]. This process gave rise to two major genetic groups of germplasm: the Andean highland group (with its center of origin and diversity in the Altiplano of Bolivia and Peru) and the lowland group (with its center of diversification in the coastal regions of central and southern Chile) [4,5].

Nowadays, the geographical distribution of the crop in South America extends from 5° N latitude to 43° S latitude (in Colombia, Ecuador, Peru, Bolivia, Argentina and Chile), and its altitudinal distribution varies from sea level to 4000 m a.s.l. [6]. Thus, due to the existence of particular adaptations from different areas throughout the Andean region, up to five ecotypes are recognized. These are associated with the corresponding sub-centers of genetic diversity and with their distinctive phenotypic and genetic characteristics: Inter-Andean valleys (Colombia, Ecuador and Peru), highlands (Peru and Bolivia), Yungas (Bolivia), Salares (Bolivia, Chile and Argentina) and coastal or sea level areas (Chile) [7,8]. Ecuador is considered a sub-center of quinoa diversification representative in the inter-Andean valleys [9,10].

According to the Food and Agriculture Organization of the United Nations (FAO), this plant genetic resource is a food with excellent nutritional properties [11], one that improves soil health by requiring less chemical fertilizer, as well as being a crop that has adapted to adverse climatic and agronomic conditions [12]. Thus, this crop offers alternative ways to produce nutritious food on marginal lands unsuitable for other conventional crops [13]. Likewise, its notable tolerance to drought and salinity makes it particularly suitable for regions outside its Andean origins [11].

The first evidence of quinoa cultivation in Ecuador dates back to 300–500 BC [14]. It was cultivated and widely used by pre-Hispanic civilizations and was replaced by cereals after the arrival of the Spanish, despite being a staple food for the Indigenous populations at the time [9]. In Ecuador, native quinoa is grown in the Andean highlands [10] and is used in a wide variety of products and foods [15]. Quinoa flour is used to make biscuits, bread, pasta, ‘empanadas,’ porridges or drinks such as ‘colada,’ while the grain is used in soups, salads or to make quinoa chocolate [15,16]. Quinoa is also important in traditional medicine, as leaves, flowers and seeds have been used by indigenous communities for their healing properties, e.g., to relieve toothache, prevent urinary infections, treat liver diseases or heal fractures [17,18].

Genetic diversity studies are very useful for assessing the biodiversity and conservation status of agricultural resources. In recent decades, a significant number of studies have been conducted to gain an understanding of the genetic diversity/structure of quinoa in its endemic range using different types of molecular markers: RAPDs [19,20], ISSRs [21,22], AFLPs [23,24] or SNPs [25,26]. Similarly, microsatellites or SSRs have also been widely used for the same purpose. The first SSR markers for quinoa were developed in the early 2000s [27,28,29]. Subsequently, these markers have been used to assess the genetic diversity in quinoa germplasm collections from different countries: USA and Peru [5], Chile [30,31], Argentina [32], Peru [33], Colombia [34,35] or Iran [36]. At present, only one study has been conducted on the characterization of the genetic diversity of Ecuadorian quinoa landraces [13]. In this study, only 84 accessions collected in the provinces of the Ecuadorian Andes were characterized using SSR markers, in order to evaluate their genetic diversity and ascertain their status for future use in breeding programs.

Data generated by genetic diversity studies of native quinoa accessions, in conjunction with agro-morphological information, are essential for the appropriate selection of promising genotypes for genetic breeding programs, as well as for the correct conservation of this valuable plant genetic resource. Accordingly, the aim of the present study was to assess the extent of genetic diversity using SSR markers in 268 accessions of quinoa landraces from the three most relevant Andean provinces in the cultivation of native quinoa (Imbabura, Cotopaxi and Chimborazo), which were collected as early as the early 1980s at two different times. This comparative analysis would provide valuable information on how farmers are managing and conserving native quinoa on their farms and would allow for the detection of possible changes in genetic diversity over time, as well as within/between provinces. Furthermore, the results obtained would also allow for an understanding of the current state of the genetic in situ conservation of this crop in the Ecuadorian highlands and its potential use for future breeding programs.

## 2. Results

Alleles obtained for each of the eight microsatellite loci analyzed in the 268 accessions of quinoa (*C. quinoa*) ordered by collection and province are shown in Supplementary Table S1. Each different allelic combination for the set of eight SSR loci constitutes a distinct genotype (G), having found 1055 different allelic combinations or genotypes (G1 to G1055) in the 268 accessions (i.e., 1340 seedlings) studied. Of all of these, 939 (89%) were unique genotypes of accession (and privates to one of the 1340 samples analyzed), and the remaining 116 were genotypes shared by two or more samples (seedlings), generally belonging to the same accession.

Only 5 of the 268 accessions showed the same genotype in the 5 seedlings/accessions analyzed (Q14: G43; Q44: G166; Q207: G883; Q241: G951; and Q268: G1055). In addition, two different genotypes were found in 18 accessions, three in 57 accessions, four in 85 accessions, and up to five different genotypes were found in 103 accessions (see Supplementary Table S1). Therefore, considering the 268 accessions separately, 1067 genotypes were found; the difference of 12 genotypes between 1067 and 1055 is due to the fact that in 12 cases two seedlings were found with the same genotype but belonged to different accessions. Thus, a total of 1067 samples have been considered for the analyses of diversity and genetic structure in the set of the 268 quinoa accessions.

### 2.1. Genetic Diversity

The results of the genetic diversity analysis carried out for the eight SSR loci in the 1067 quinoa samples considered are showed in Table 1. The alleles and their frequencies found in each of the eight SSR loci analyzed in the set of 1067 samples, without considering the collection or the province of origin, are shown in Supplementary Table S2. In addition, Supplementary Table S3 shows the genotypes detected, and their frequencies, in each of the eight loci.

The number of alleles (N_a_) ranged from 11 (locus 5_QAAT076) to 24 (locus 7_QAAT100), with a mean of 15.5 per locus and a cumulative of 124 alleles considering all loci (Table 1). Two to four alleles were found with a frequency greater than 10% at all loci, and only in five of them was there an allele with a frequency > 0.3 (see Supplementary Table S2). The number of genotypes (N_g_) varied between 30 (locus 8_QAAT106) and 96 (locus 7_QAAT100), with a mean of 52.9 per locus and a cumulative of 423 genotypes for all loci (Table 1). Only one to three genotypes with a frequency greater than 10% were found in each locus, and in seven of them there was only one genotype with a frequency > 0.2, coinciding with the homozygous genotype for the corresponding major allele (see Supplementary Tables S2 and S3).

The effective number of alleles (N_e_) presented an average value of 4.673 per locus, varying between 2.980 for locus 5_QAAT076 and 7.787 for locus 7_QAAT100 (Table 1). The average values of observed (*H_o_*) and expected (*H_e_*) heterozygosity were 0.314 and 0.763, respectively. Maximum values were obtained in both cases for 7_QAAT100 (*H_o_* = 0.348; *H_e_* = 0.872), and minimum in *H_o_* for 8_QAAT106 (0.262) and *H_e_* for 5_QAAT076 (0.664) (Table 1). The average Shannon information index (I) for the set of eight loci was 1.821, ranging from 1.503 (5_QAAT076) to 2.375 (4_AHAAC011) (Table 1). Finally, the minimum and maximum values of the discrimination power (D) were again for 5_QAAT076 (79.7%) and 7_QAAT100 (93.4%), respectively; with a cumulative probability for the set of all loci of practically 1 (Table 1).

The results of the genetic diversity analysis comparing different collections and provinces are shown in Table 2. The average number of allelic combinations or genotypes (G) found per collection and province was notably high (>70) and evidently associated with the number of accessions analyzed in each province and collection. On the other hand, the average number of alleles (N_a_) detected was slightly higher in the accessions of Collection B (10.46) than in those of Collection A (9.71), and the highest value was observed in Collection B from Chimborazo (12.38; Table 2), probably due to the greater number of accessions analyzed in this case. The effective number of alleles (N_e_), the expected heterozygosity (*H_e_*) and the Shannon information index (I) are parameters commonly used to assess the extent of genetic diversity. The mean values of these parameters were slightly higher for the set of accessions from Collection A, but no significant differences were detected between the two collections or among provinces (Table 2).

However, the mean values of observed heterozygosity (*H_o_*) were significantly (*p* < 0.05) higher in Collection B (0.281) than in Collection A (0.226). Although highly significant differences (*p* < 0.001) were only detected between both collections in the province of Chimborazo (0.383 for Collection B; 0.158 for Collection A). In Cotopaxi province, mean *H_o_* values were similar in both collections; and in Imbabura province, these values were higher in the accessions of Collection A (Table 2). In relation to these data, it is noteworthy that 256 seedlings out of the 1067 considered in the analysis (24%) showed all eight loci in homozygosity, and the remaining 811 seedlings (76%) showed at least one locus in heterozygosity (see Supplementary Table S1). On the other hand, it is worth mentioning that 30 unique or private collection/province alleles were found: 13 in Collection A (2 for Chimborazo province, 6 for Cotopaxi and 5 for Imbabura) and 17 in Collection B (14 for Chimborazo, 1 for Cotopaxi and 2 for Imbabura).

Table 3 shows Nei’s genetic distances matrix among the six groups of accessions analyzed (two collections x three provinces). In Collection A, the greatest genetic distance was obtained between the provinces of Chimborazo and Cotopaxi (0.332), while Imbabura presented lower and similar values with the other two provinces (0.200 with Chimborazo and 0.201 with Cotopaxi). In Collection B, the Chimborazo accessions showed a greater genetic distance with those from the other provinces (0.207 with Cotopaxi and 0.221 with Imbabura), which were genetically more similar to each other (0.113) (Table 3). When accessions collected in the same province but at different times were compared, the genetic distance value between both collections in Chimborazo was lower (0.069) than in Cotopaxi (0.159) and Imbabura (0.122) (Table 3).

The genetic diversity analysis was completed with an analysis of molecular variance (AMOVA; see Supplementary Table S4). In all cases and hierarchical levels, the estimates obtained for the different molecular variance components were highly significant (*p* < 0.0001). When both collections were analyzed together, it was observed that the percentage of molecular variance due to differences among collections was practically null (0.5%), whereas 6.1% of the total variance was due to differences among provinces, and the remaining 93.4% to differences among and within samples/accessions (53.3% and 40.1%, respectively).

When analyzing the results for each collection, in both cases the highest percentage of the variance (about 94%) was again due to differences among and within samples, and only about 6% of the variance could be explained by differences between provinces (see Supplementary Table S4).

### 2.2. Genetic Structure

**Figure 1:**
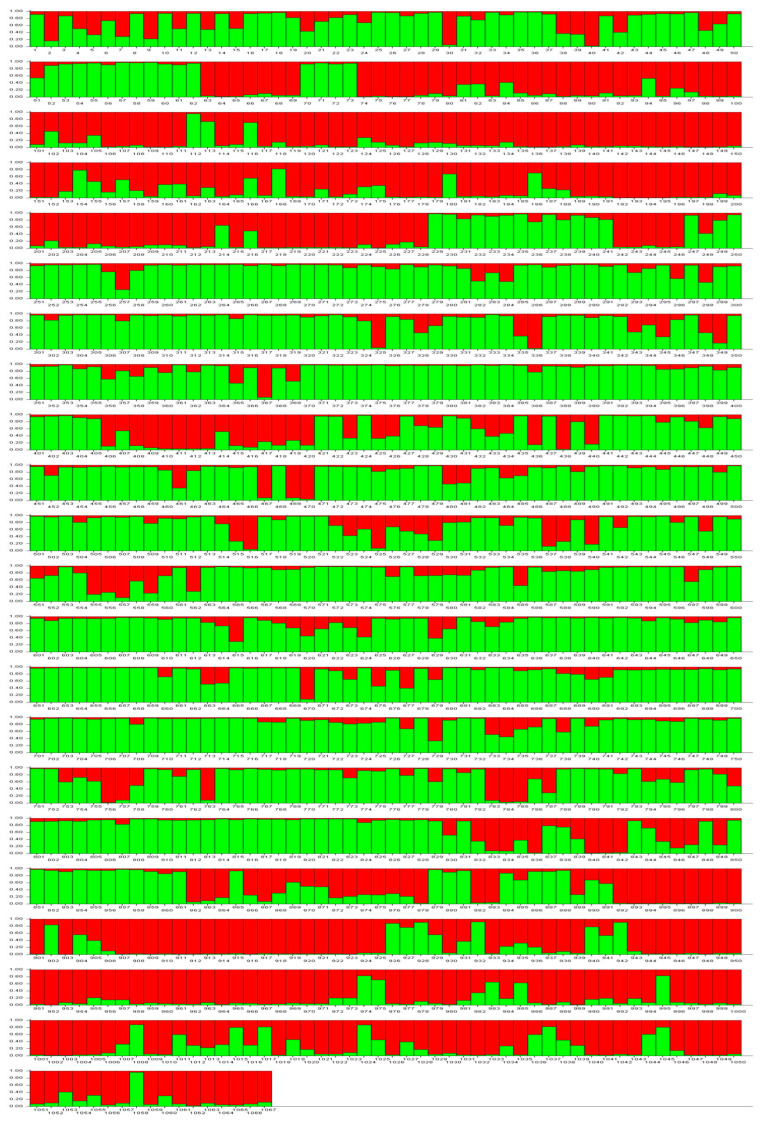
STRUCTURE assignment model for the 1067 quinoa samples when K = 2 genetic clusters and using the data of eight simple sequence repeat (SSR) markers (Cluster 1 in red and Cluster 2 in green). Membership coefficients (Q’s) are depicted vertically for each sample. Sample numbers coincide with the number assigned in Supplementary Table S1.

The STRUCTURE analysis of 1067 quinoa samples using the data of eight SSR loci showed that the best estimate of the optimal number of genetically different groups was obtained for the value of K = 2 (see Supplementary Figure S1), i.e., the detected genetic diversity seems to be structured in two clusters of samples/accessions. To assign each of the 1067 samples to one of the two clusters, it was considered that they had a membership coefficient Q > 0.6. Figure 1 graphically shows the membership coefficients depicted vertically for each of the 1067 samples analyzed. Thus, 390 (36.6%) samples were assigned to Cluster 1, 616 (57.7%) to Cluster 2, and the remaining 61 (5.7%) could not be assigned to either cluster (Table 4).

The genetic structure analysis in the quinoa accessions studied showed that about 81% (315/390) of the samples assigned to Cluster 1 corresponded to accessions collected in the provinces of Cotopaxi (73 samples from Collection A and 61 from Collection B) and Imbabura (67 samples from Collection A and 114 from Collection B); while about 90% of the samples in Cluster 2 belonged to accessions collected in the province of Chimborazo (50 samples from Collection A and 507 from Collection B) (Table 4).

Results of the genetic diversity comparative analysis for the two sample groups defined by STRUCTURE analysis (Cluster 1 and Cluster 2) are shown in Table 5. The genetic parameters N_a_, N_e_, *H_e_*, and I did not show significant differences between both groups of samples, although in all of them greater values were obtained for Cluster 1. Conversely, significant differences (*p* < 0.05) were detected between the two clusters for the observed heterozygosity (*H_o_*), with Cluster 2 showing significantly higher values (Table 5). On the other hand, 41 unique or cluster-private alleles were found, 26 of them in samples assigned to Cluster 1 and the remaining 15 specific to samples from Cluster 2. The results of the analysis of molecular variance in the two clusters defined using the STRUCTURE program are in Supplementary Table S5. The AMOVA showed that 9.5% of the total variance was due to differences among clusters, and 90.5% to differences among (51.6%) and within (38.9%) samples/accessions. Likewise, the value of Nei’s genetic distance between the two clusters was relatively low (0.337).

### 2.3. Genetic Relationships Among Genotypes

**Figure 2:**
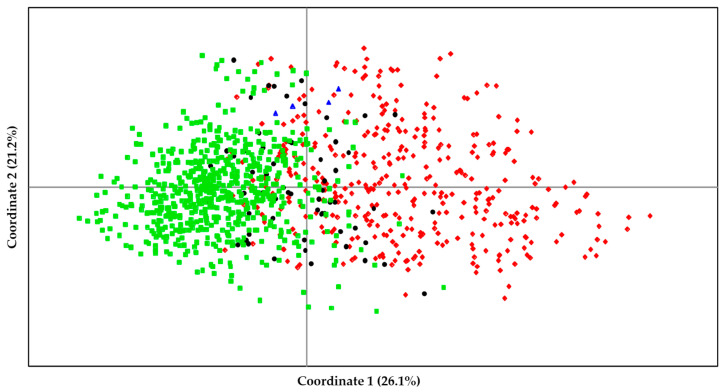
Principal coordinates analysis (PCoA) of the 1055 different genotypes found in all of the 268 quinoa accessions, and the 4 genotypes found in the improved INIAP–Tunkahuan variety. Percentages of variation explained by each of the first two coordinates are indicated. The color of each genotype matches that assigned to the cluster in the STRUCTURE analysis: Cluster 1 in red (rhombus), Cluster 2 in green (squares), and unassigned samples in black (circles). Genotypes of the INIAP–Tunkahuan variety are shown in blue triangles.

In order to understand the genetic relationships among the 1055 different genotypes that were found in the set of 268 quinoa accessions (1340 samples) studied, a principal coordinate analysis was carried out (Figure 2), in which the four genotypes found in the commercial variety INIAP–Tunkahuan were also included as a reference (G_T1, G_T2, G_T3, and G_T4; see Supplementary Table S1). The percentage of cumulated variation explained by the first two coordinates was 47.3% (26.1% for Coordinate 1 and 21.2% for Coordinate 2). The first coordinate separated the majority of the genotypes associated with each samples cluster into two groups, leaving the genotypes not assigned to a cluster mixed and dispersed in between (Figure 2) so that the results of the STRUCTURE analysis would be supported by the PCoA results. The four genotypes of the variety INIAP–Tunkahuan were located between the two groups of genotypes found in the accessions of quinoa landraces (Figure 2). The Nei’s genetic distance between the genotypes of the INIAP–Tunkahuan variety and each of the groups of genotypes associated with Clusters 1 and 2 was greater than the genetic distance between both clusters. Furthermore, the genetic distance with the genotypes of Cluster 2 was greater than with those of Cluster 1 (0.551 vs. 0.404).

## 3. Discussion

### 3.1. Genetic Diversity

Quinoa grown by local farmers in the Ecuadorian Andean region may contain native germplasm with some interesting phenotypes/genotypes for future breeding programs [13,37]. The study of accessions collected during two different time periods, separated by about four decades, has allowed us to determine their genetic diversity and current genetic conservation status, and has also provided information about the changes in genetic diversity that have occurred in recent decades. In the present study, we found high levels of genetic diversity using microsatellite markers in landraces of quinoa from the Andean region of Ecuador. These molecular results agree with the high phenotypic diversity observed in the previous agro-morphological characterization of these landraces [37], as well as with other studies that have also shown the existence of an extensive phenotypic variation in the Ecuadorian quinoa germplasm [10,38].

On the other hand, it was surprising that 1055 different genotypes were detected in the 1340 samples analyzed (5 seedlings × 268 accessions), also revealing the existence of a high intra-accession diversity. No studies published to date have reported within-accession variability of this species. It is only Manjarres-Hernández and Morillo [35] that have indicated that they separately evaluated three individuals per accession, and pointed out that all individuals from the same accession were highly homogeneous.

The values obtained for the genetic diversity parameters reveal a high diversity in the Andean accessions analyzed, similar to those found in other studies of cultivated quinoa from different South American countries, such as Bolivia, Chile, Argentina, Peru, and Colombia [5,30,31,32,33,35,39], and even from Ecuador [13]. Some of these studies indicate that the highest diversity values (e.g., *H_e_* > 0.7) have been obtained when studying germplasm collected in the Andean Altiplano [31,39], supporting the hypothesis that this region would be the center of origin and the greatest diversity of quinoa [4,5,40]. As suggested by Salazar et al. [13], our results would also support the previous hypothesis of Christensen et al. [5] on the possible origin of quinoas from the Ecuadorian Andean region in germplasm from the Altiplano of Bolivia and Peru, following a process of differentiation and adaptation to local conditions through domestication by generations of farmers. Similarly, Manjarres-Hernández and Morillo [35] have found high levels of genetic diversity in Colombian accessions and have also suggested that this could be due to their origin in germplasm from the Andean Altiplano.

In terms of observed heterozygosity (*H_o_*), relatively high values were also obtained (0.262 to 0.348; mean = 0.314) for a predominantly self-pollinated species, whose estimated cross-pollination percentages ranged from 10 to 17 [41,42]. However, quinoa accessions cultivated in different municipalities of Colombia showed higher mean *H_o_* values (>0.5) [34,35,43], becoming very high (*H_o_* mean = 0.790) in quinoa varieties cultivated in the Peruvian Altiplano [33]. On the other hand, 76% of the genotypes found in the analyzed samples showed at least one locus in heterozygosity. These high values of genetic heterogeneity contrast with the low values found by Salazar et al. [13], who found only 16% of heterozygous genotypes at any given SSR locus in 84 quinoa accessions collected from seven provinces of the Ecuadorian highlands. Such high levels of genetic heterogeneity could be explained by the existence of “involuntary” crossing processes in the field with genotypes from other regions, which could have been obtained by the farmer through seed exchange in local markets [13], and in more recent decades also in seed exchange fairs or in centers for bioknowledge and agricultural development that emerged in the late 1990s [38,44].

Therefore, the high diversity values obtained in the present study could indicate that the quinoa landraces in the Andean region of Ecuador have been selected over generations by the farmers themselves, without any control over possible seed mixing or even pollination, thus facilitating gene flow between different genotypes grown in the same planting area [26]. Kolano et al. [45] also indicate that high percentages of polymorphic loci and high heterozygosity values can be attributed both to the allotetraploid origin of *C. quinoa* and the natural gene flow present in the germplasm of quinoa.

Regarding the efficiency of the eight SSR loci used, it should be noted that they showed a high theoretical discrimination power (practically 1), making it possible to distinguish 1055 different genotypes in the 1340 samples (from 268 accessions) analyzed. In particular, four of the eight loci used (1_QAAT022, 3_QAAT050, 6_QAAT097, and 7_QAAT100) were shown to be very informative and efficient in detecting the diversity existing in these quinoa accessions. These same loci were also very efficient in other studies in which genetic diversity was analyzed in quinoa accessions with different origins [30,32,35]; thus, we believe that they should be recommended for future studies of genetic diversity in quinoa germplasm.

When comparing different collections and provinces, the number of different genotypes detected in each province for each of the two collections was quite high and, as expected, was correlated with the number of accessions analyzed in each province and collection. In general, although the number of accessions evaluated was lower in Collection A, genetic diversity parameters were found to be slightly higher in those accessions collected in the 1980s than in those collected more recently, but no significant differences were detected.

These results were confirmed by the analysis of molecular variance, in which the percentage of variance due to differences among collections was practically nil (0.5%), and among provinces only 5.7% (Collection A) and 6.2% (Collection B). This seems to indicate that the high diversity detected would already be present in the gene pool of ancestrally cultivated quinoa accessions in the Andean region of Ecuador, and that the processes of exchange and mixing of seeds from different areas of the Ecuadorian highlands do not appear to have quantitatively affected the diversity of this gene pool, although it may have been qualitatively modified in a particular way in each of the provinces due to the different behavior of farmers in each area or province with respect to the management and conservation of these traditional varieties [37,44].

Significant differences were found between the two collections only for the mean values of observed heterozygosity, which were due exclusively to accessions collected in the Ecuadorian province of Chimborazo. These results could be explained by the fact that two of the six recognized centers of bioknowledge and agricultural development, which have facilitated an intensive seed exchange between farmers in recent decades, are in Ecuador [44].

However, in the province of Chimborazo, only the so-called variety or ecotype “Chimborazo” is theoretically cultivated [46], which in fact refers to a mixture of landraces that have been ancestrally cultivated exclusively in this province [9]. Therefore, the intensive exchange of seeds in Chimborazo tends to be mainly within the province. This would also be supported by the low genetic distance value obtained for the two collections in this province (0.069), so that the degree of introgression of germplasm from other areas/provinces or even improved varieties must have been relatively limited. Likewise, farmers in the province of Chimborazo have tried to preserve native quinoa as best they can, making their own selection processes according to their needs and the market. This means that the Chimborazo variety is now widely accepted both nationally and internationally, in particular because it has traditionally been grown using agroecological or organic production systems [47].

Regarding the provinces of Imbabura and Cotopaxi, the genetic distances between collections were higher than those found in Chimborazo (0.122 for Imbabura and 0.159 for Cotopaxi), which could be due to the fact that, during the last decades, farmers in these provinces have largely ceased cultivating native varieties in favor of improved varieties that are well adapted to the area and with higher productivity [9,47], so that the degree of possible introgression of germplasm from other areas/provinces or of the improved varieties themselves into the traditional varieties may have been greater than in Chimborazo.

Currently, quinoa landraces are grown as a minority and for self-consumption of the farming communities in Imbabura and Cotopaxi [9], while most of the quinoa grown for commercial purposes in these provinces corresponds to the improved INIAP–Tunkahuan variety [47]. This commercial variety was released in the early 1990s [48], in order to increase quinoa production and consumption nationwide [9], and its production has since been promoted through the commercialization of certified seed in Ecuador [9,13].

On the other hand, the AMOVA results show that the highest percentage of the molecular variance was due to differences among (53.3%) and within (40.1%) the accessions collected in each of the provinces. These data once again confirm the high level of diversity that exists, both inter- and intra-accession, in quinoa landraces grown in the Ecuadorian highlands. Morillo et al. [34], who analyzed the genetic diversity of accessions collected in different municipalities of the department of Boyacá, also found high values of molecular variance due to differences among and within accessions (82% compared to 93.4% in our case). However, in the evaluation of the genetic diversity and structure of germplasm from northwestern Argentina [32,49], the AMOVA analyses showed strong differentiation among populations and low values of the variance due to differences among (26%) and within (16%) accessions, indicating that it could reflect the influence of cultural practices used by farmers over long periods.

### 3.2. Genetic Structure and Relationships Among Genotypes

The assignment of the samples to each of the two clusters identified by the STRUCTURE analysis seems to be apparently related to their province of origin, regardless of the time of collection, as 81% of the samples assigned to Cluster 1 corresponded to accessions collected in the provinces of Cotopaxi and Imbabura, and 90% of the samples in Cluster 2 belonged to accessions collected in the province of Chimborazo. On the contrary, when the genetic diversity was evaluated in accessions collected from different Andean provinces of Ecuador [13] or in germplasm from different municipalities in Colombia [34,35], it was found that the differentiated genetic groups did not show any association with the geographical origin of the accessions.

In order to explain our results, it is necessary to recall that farmers in the province of Chimborazo predominantly cultivate the variety “Chimborazo” [9,47]. Thus, it is very likely that the introgression processes of germplasm from other areas of the Andean region were uncommon, resulting in the gene pool of most of the accessions in this province gradually differentiating from that of the other Andean provinces over the recent decades and adapting to the specific environmental and cultural conditions of Chimborazo [9].

On the other hand, the greater similarity of the gene pool detected between the provinces of Imbabura and Cotopaxi could be explained because, in both provinces in which the cultivation of quinoa landraces is a minority, there is a high level of seed exchange, usually with other areas or provinces of the Ecuadorian highlands [9,44]. This high seed exchange, in many cases through informal networks connecting farmers from different altitudes and latitudes, occurs with most Andean crops in Ecuador [13] and is also a very common practice in Colombian quinoa-growing areas [35].

Regarding the genetic differences between the two clusters, the analysis of molecular variance showed that only 9.5% of the total variance was due to differences between the two genetic groups, compared with the values of around 20% found for differentiated genetic clusters in germplasm collected in different municipalities of Colombia [35,43]. Moreover, no significant differences were detected for the main parameters of genetic diversity (N_a_, N_e_, *H_e_* and I). However, a relatively high Nei’s genetic distance value was obtained between the two clusters (0.337), indicating that they have somewhat different genetic diversity, at least from a qualitative point of view. This is also supported by the presence of an important number of unique or private alleles in each cluster (26 for Cluster 1 and 15 for Cluster 2).

Salazar et al. [13] differentiated three distinct genetic clusters in modern accessions collected in the Andean provinces of Ecuador, each with a highly heterogeneous geographical composition. The lack of a clear geographical pattern in the genetic structure led them to hypothesize that each cluster constitutes an independent ancestral genetic lineage, representing either ancestral landraces or modern bred varieties disseminated throughout the Ecuadorian Andean region. In our case, the two differentiated genetic clusters do seem to show a certain geographical structure, so that this would not support the idea of associating the clusters with distinct ancestral genetic lineages, but rather with a different way of managing and conserving quinoa landraces in the different Andean provinces, together with the aforementioned common practice of seed exchange and mixing, as well as the possible introgression of commercial varieties in some cases.

On the other hand, the principal coordinates analysis again showed a distribution of the genotypes in two groups that coincided with the assignment of these genotypes to Clusters 1 and 2. The genotypes of Cluster 2 were closer to each other than those of Cluster 1, so that this greater dispersion of genotypes of Cluster 1 would confirm the slightly higher levels of diversity that were found in this cluster. Regarding the genotypes found in the INIAP–Tunkahuan variety, they showed greater genetic distances with the two genotype clusters than those obtained between the two clusters. This could indicate that, although there may be some introgression of this improved variety into traditional varieties, the level of differentiation with them is still relatively high. Moreover, as previously suggested by Salazar et al. [13], our data also seem to point out that, during the dissemination of this commercial variety through different farmer seed exchange networks, its genetic homogeneity has been lost by outcrossing with other quinoa germplasms cultivated in this region.

Genetic diversity studies of local varieties, together with their phenotypic evaluation, are essential for the appropriate management and conservation of these valuable plant resources [13,37]. In this study, we have provided important information on the current genetic conservation status of quinoa landraces from the Ecuadorian highlands, as well as on the assessment of their variation over the last four decades.

The loss of biodiversity in underutilized traditional Andean crops could be a major problem for future breeding programs seeking to take advantage of their agronomic resilience and nutritional quality [4]. In our study, we were able to show that no significant loss of genetic diversity in quinoa landraces has been detected in the last four decades, at least quantitatively. Thus, indigenous farming communities in the Ecuadorian Andean region seem to have maintained their agrobiodiversity reasonably well, preserving their quinoa landraces as an essential part of their family heritage. Tapia and Rosas [50] pointed out that the in situ conservation of Ecuadorian Andean crops has been primarily undertaken by the indigenous communities. However, in recent years, the conservation of this agrobiodiversity has been led by farmers in collaboration with non-governmental organizations (NGOs), which have promoted the seed exchange processes, the local fairs, the strengthening of conservationist farmers and the increase in ancestral practices for the management and conservation of local diversity [51]. Furthermore, the molecular characterization carried out in the present study could be taken into account in future breeding programs, as well as in the management of the native quinoa accessions collection conserved in the INIAP germplasm bank.

## 4. Materials and Methods

### 4.1. Plant Material

**Figure 3:**
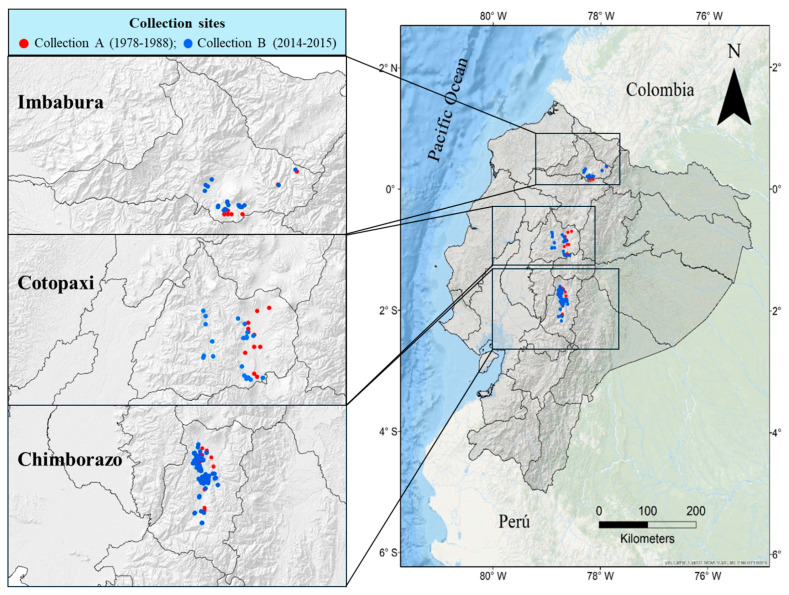
Geographical map of Ecuador indicating the collection sites of 268 quinoa accessions in the provinces of Imbabura, Cotopaxi and Chimborazo during 1978–1988 (Collection A; 61 accessions) and 2014–2015 (Collection B; 207 accessions).

Two hundred and sixty-eight quinoa (*Chenopodium quinoa* Willd.) landrace accessions were analyzed. These accessions were collected at two different times (“Collection A” was carried out between 1981 and 1986 and “Collection B” took place in 2014 and 2015), both in three provinces of the Ecuadorian Andean region that are representative of this crop in terms of the cultivated area, production, consumption and economic importance [9,52]: Chimborazo, Cotopaxi and Imbabura. A total of 109 accessions were collected at the first collection phase (Collection A), of which only the 61 with complete passport data were selected: 20 from Chimborazo (Q1 to Q20), 20 from Cotopaxi (Q21 to Q40) and 21 from Imbabura (Q41 to Q61). In Collection B, 207 accessions were collected: 143 from Chimborazo (Q62 to Q204), 28 from Cotopaxi (Q205 to Q232) and 36 from Imbabura (Q233 to Q268) (Figure 3 and Supplementary Table S1). These accessions were donated to be preserved in the Germplasm Bank (GB) of the National Institute for Agricultural Research (INIAP), located in the province of Pichincha, Ecuador, with the exception of six accessions that were not donated by farmers for conservation in the GB. The INIAP–GB codes as well as the analysis codes used in this study (Q1 to Q268) are indicated in Supplementary Table S1, and all passport data can be found in Delgado et al. [37]. Thus, to analyze the genetic diversity among collections and among provinces of the same collection or different collections, six groups of accessions were considered: Chimborazo, Cotopaxi, and Imbabura from Collection A, and Chimborazo, Cotopaxi, and Cañar from Collection B. The commercial variety INIAP–Tunkahuan was also included in our analysis and was used as a reference to detect possible introgression of this improved germplasm in the quinoa landraces. The high number of accessions collected through 2014 and 2015 in the province of Chimborazo is due to the fact that it was possible to prospect in a greater number of sites, as the cultivation of native quinoa here is much more abundant than in the rest of the Andean provinces. In addition, the farmers of this province usually separate the cultivation of their quinoa landraces according to the color of the panicle (lighter colors—yellow or green—on the one hand and darker colors—pink, purple or red—on the other [9]) so that in most sites, two accessions were collected per donor farmer.

### 4.2. DNA Extraction and Microsatellite Genotyping

Leaf material was collected from five seedlings per accession (and for variety INIAP–Tunkahuan) grown under greenhouse conditions during 20–25 days and was stored at –80 °C until DNA extraction. Total genomic DNA was extracted from about 100 mg of leaf tissue following the protocol supplied in the “NucleoSpin^®^ Plant II Kit” (Macherey-Nagel GmbH & Co. KG, Düren, Germany). Quantification of the extracted DNA was carried out by visual comparison with known concentrations of lambda DNA on 1.2% (*w/v*) agarose gels, and a working solution of DNA (approx. 10 ng/µL) was made.

Forty SSR loci were shortlisted among the more than 400 polymorphic microsatellite markers previously developed and validated by Mason et al. [28] and Jarvis et al. [29], taking into account the reported values of expected heterozygosity (*H_e_* > 0.7) and the number of alleles per locus (>5), as well as their efficiency in other studies, in order to analyze the genetic diversity in quinoa germplasm. In a preliminary analysis, the 40 SSR loci were screened using 30 representative samples of our quinoa landraces (five samples of each province/collection). All of these SSR markers only amplified one locus each [28,29]. Finally, eight of the 40 SSR markers were selected for use over the 268 accessions (1340 samples), because they were the ones that presented analyzable amplified products, and in which greater polymorphism was detected in the preliminary analysis. Moreover, at least six of these are located in different linkage groups [29]. Table 6 summarizes forward and reverse primer sequences, linkage groups, repeat motifs, and fluorescent dyes used to label at 5′ ends of the forward primer of each pair, and annealing temperatures for eight selected microsatellite loci. All of the loci that were finally selected belonged to those previously described by Mason et al. [28].

Polymerase chain reactions (PCRs) were performed in 15 µL of final volume containing about 20 ng of template DNA, 0.5 µM of each of the forward and reverse primers, 0.15 mM of each deoxynucleotide triphosphate (dNTP), 2 mM of MgCl2, and 1 U of Tth DNA polymerase in 1X of the manufacturer’s reaction buffer (BIOTOOLS, B&M Labs, Madrid, Spain). DNA amplifications were carried out in a PTC-100 thermalcycler (MJ Research, Inc., Waltham, MA, USA) with heated lid, using the same protocol for all microsatellite loci: an initial denaturalization step of 4 min at 94 °C, followed by 35 cycles of 45 s at 94 °C, 1 min at 50 or 52 °C (depending of the primer pair; see Table 1) and 1 min 10 s at 72 °C, with a final extension step of 72 °C for 5 min. Amplified products were resolved by capillary electrophoresis using an ABI PRISM^®^ 3730 DNA analyzer (Applied Biosystems, Foster City, CA, USA). GeneScan-500 LIZ (Applied Biosystems, Foster City, CA, USA) was used as an internal size standard. Alleles were scored using Peak Scanner™ software version 1.0 (Applied Biosystems, Foster City, CA, USA).

### 4.3. Data Analysis

Genetic diversity parameters for the eight microsatellite loci were calculated based on data from the set of the 268 accessions studied. Thus, allele number (N_a_) and their frequencies, effective allele number (N_e_), number of observed genotypes (N_g_), observed (*H_o_*) and expected (*H_e_*) heterozygosity, and Shannon’s information index (I) were calculated for each locus using GenAlEx software version 6.5 [53]. Furthermore, the discrimination power (D; [54,55]) is an estimate of the probability that two randomly sampled accessions could be distinguished by their microsatellite profiles and was calculated for each locus as D = 1 − C, where C is the probability of coincidence, i.e., two samples match by chance at one locus (C = ΣPi2, and Pi is the frequency of different genotypes observed at that locus). The discrimination power for all loci combined (m = 8) was calculated as DT = 1 − CT, where CT = ΠCm, and represents the cumulative probability of coincidence for all loci.

On the other hand, to analyze the genetic diversity among collections and among provinces of the same collection or different collections, data from the overall microsatellite loci analyzed were used to calculate the number of allelic combinations over all loci (or genotypes (G)) in each of the accession groups. Other parameters, such as the mean over all loci of N_a_, N_e_, *H_o_*, *H_e_*, and I in each accession group, were also calculated using GenAlEx software [53]. To detect significant differences in these parameters among different accession groups, the corresponding analysis of variance (ANOVA) was performed, and means were compared using a Fisher’s least significant difference (LSD) test [56]. On the other hand, Nei’s genetic distances [57] among the different groups of accessions that were considered were also calculated. Additionally, in order to determine the significance of the partitioning of genetic diversity among and within collections/provinces/accessions, an analysis of molecular variance (AMOVA [58]) was conducted using the GenAlEx package. Levels of significance of variance component estimates were computed by non-parametric permutational procedures using 10,000 random permutations.

In addition, STRUCTURE software version 2.3.4 [59] was used to identify genetic groups in the quinoa samples. This Bayesian approach uses no a priori classification and assigns samples to K genetic clusters based on the allele frequencies at each locus. The range of possible groups (K) tested varied from 1 to 10. The estimate of the most likely number of genetic groups was performed following the procedure of Evanno et al. [60]. The program settings used were the admixture ancestry and correlated allele frequency models. The degree of admixture (alpha) was inferred from the data, and lambda, the parameter of the distribution of allelic frequencies, was set to 1 [37,61]. The program was run 20 independent times for each K value. In each run, a burn-in period of 10,000 iterations, and 100,000 post-burning Markov chain Monte Carlo (MCMC) simulations were carried out. Finally, among the 20 runs performed for the estimated optimal K value, the run with the least negative log–likelihood value was used to obtain the assignation membership coefficients (Q’s) for each sample in each of the K-inferred groups [62].

Likewise, a comparative analysis of the genetic diversity in the K-obtained groups of samples was carried out. Thus, for each group, the mean values of N_a_, N_e_, *H_o_*, *H_e_*, and I were calculated. To detect significant differences among groups, an ANOVA and a Fisher’s LSD test were carried out. Nei’s genetic distances among groups of accessions were also calculated. In addition, the partition of genetic diversity among groups, and among and within samples of each group, was estimated by an AMOVA, estimating the variance components and their level of significance using a non-parametric procedure with 10,000 permutations. All of these analyses were conducted using GenAlEx package. Furthermore, a principal coordinate analysis (PCoA) of the different genotypes found in the 268 accessions was carried out, to determine the genetic relationships among them. The genotypes found in the commercial variety INIAP–Tunkahuan were included as a reference, in order to determine their relationship with respect to the genotypes of landraces. GenAlEx package was used to generate the matrix of squared distances between pairs of genotypes that were used to carry out the principal coordinate analysis.

## 5. Conclusions

High genetic diversity was found in quinoa landraces from the Ecuadorian Andean region. This could be due to a possible origin in the germplasm from the Andean Altiplano. No significant differences were found in the main parameters of genetic diversity when comparing different collections and provinces, indicating that the high diversity detected would already be present in the gene pool of quinoas traditionally cultivated in the Andean region of Ecuador. Significant differences were found only for the mean values of observed heterozygosity between accessions collected at different times in the province of Chimborazo. This is probably due to an intensive seed exchange within the province over recent decades, where only the so-called “Chimborazo” variety is cultivated. However, in the provinces of Imbabura and Cotopaxi, there appears to have been a greater degree of introgression of germplasm from other areas/provinces or of the improved varieties, which has led to a loss of genetic identity in the landraces ancestrally cultivated in these provinces. The two genetic clusters identified seem to be related to the province of origin, regardless of the time of collection. This may be due to the different ways in which quinoa landraces are managed and conserved in the different Andean provinces. Finally, there has been no significant quantitative loss of genetic diversity detected in quinoa landraces over the last four decades. We can therefore say that indigenous farming communities of the Ecuadorian Andean region appear to be preserving their quinoa germplasm reasonably well.

## Figures and Tables

**Table 1:** Genetic diversity parameters calculated for each of the eight microsatellite loci analyzed in 1067 quinoa samples. N_a_: number of alleles, N_e_: effective number of alleles, N_g_: number of genotypes, *H_o_*: observed heterozygosity, *H_e_*: expected heterozygosity, I: Shannon information index, C: probability of coincidence, and D: discrimination power.

No._Locus	N_a_	N_e_	N_g_	*H_o_*	*H_e_*	I	C	D
1_QAAT022	19	4.725	67	0.298	0.788	1.987	0.127	0.873
2_QAAT024	14	3.647	36	0.312	0.726	1.636	0.159	0.841
3_QAAT050	16	6.621	69	0.347	0.849	2.144	0.072	0.928
4_QAAT070	12	3.756	31	0.291	0.734	1.566	0.159	0.841
5_QAAT076	11	2.980	35	0.318	0.664	1.503	0.203	0.797
6_QAAT097	16	4.640	59	0.339	0.784	1.852	0.112	0.888
7_QAAT100	24	7.787	96	0.348	0.872	2.375	0.066	0.934
8_QAAT106	12	3.226	30	0.262	0.690	1.509	0.201	0.799
Mean	15.5	4.673	52.9	0.314	0.763	1.821	-	-
Cumulative	124	-	423	-	-	-	0.0000000699	0.9999999301

**Table 2:** Genetic diversity parameters calculated for provinces of Chimborazo, Cotopaxi, and Imbabura in accessions collected between 1978 and 1988 (Collection A) and accessions collected in 2014 and 2015 (Collection B). G: number of genotypes (allelic combinations). Average values of allele number (N_a_), effective number of alleles (N_e_), observed heterozygosity (*H_o_*), expected heterozygosity (*H_e_*), and Shannon information index (I). Values showing significant differences (* *p* < 0.05; *** *p* < 0.001) between collections are indicated in bold.

Provinces (Collection)	G	N_a_	N_e_	*H_o_*	*H_e_*	I
Chimborazo (A)	72	8.88	4.345	0.158 ***	0.725	1.622
Cotopaxi (A)	**79**	10.38	4.816	0.261	0.771	1.828
Imbabura (A)	77	9.88	4.126	0.258	0.750	1.693
*Mean* Collection A	*76.0*	*9.71*	*4.429*	0.226 *	*0.748*	*1.714*
Chimborazo (B)	597	12.38	4.222	0.383 ***	0.712	1.649
Cotopaxi (B)	98	10.00	5.078	0.268	0.791	1.825
Imbabura (B)	132	9.00	3.861	0.193	0.724	1.576
*Mean* Collection B	*275.7*	*10.46*	*4.387*	0.281 *	*0.742*	*1.683*

**Table 3:** Matrix of Nei’s genetic distances among the six groups of accessions analyzed (two collections, A and B, x three provinces: Chimborazo, Cotopaxi, and Imbabura).

	Chimborazo_A	Cotopaxi_A	Imbabura_A	Chimborazo_B	Cotopaxi_B	Imbabura_B
**Chimborazo_A**	---					
**Cotopaxi_A**	0.332	---				
**Imbabura_A**	0.200	0.201	---			
**Chimborazo_B**	0.069	0.451	0.265	---		
**Cotoplaxi_B**	0.214	0.159	0.207	0.207	---	
**Imbabura_B**	0.238	0.280	0.122	0.221	0.113	---

**Table 4:** Assignment of the 1067 quinoa samples from province (Chimborazo, Cotopaxi and Imbabura) and by collection (A and B) to each of the two clusters defined by the STRUCTURE program, with a membership coefficient Q > 0.6. Percentages of assigned samples greater than 60% are shown in bold.

Provinces (Collection)	Cluster 1	Cluster 2	Not Assigned
Chimborazo (A)	15 (20.5%)	**50 (68.5%)**	8 (11.0%)
Cotopaxi (A)	**73 (92.4%)**	3 (3.8%)	3 (3.8%)
Imbabura (A)	**67 (87.0%)**	6 (7.8%)	4 (5.2%)
Chimborazo (B)	60 (10.0%)	**507 (84.2%)**	35 (5.8%)
Cotopaxi (B)	**61 (61.6%)**	32 (32.3%)	6 (6.1%)
Imbabura (B)	**114 (83.2%)**	18 (13.1%)	5 (3.6%)
Total	390 (36.6%)	616 (57.7%)	61(5.7%)

**Table 5:** Genetic diversity parameters calculated for accessions of Clusters 1 and 2 defined by STRUCTURE analysis. Average values of allele number (N_a_), effective number of alleles (N_e_), observed heterozygosity (*H_o_*), expected heterozygosity (*H_e_*), and Shannon information index (I). For each parameter (data column), different letters indicate significant differences (*p* < 0.05).

	N_a_	N_e_	*H_o_*	*H_e_*	I
Cluster 1	13.63 a	4.672 a	0.233 a	0.776 a	1.832 a
Cluster 2	12.25 a	4.144 a	0.364 b	0.695 a	1.591 a
*Mean*	*12.94*	*4.408*	*0.298*	*0.736*	*1.711*

**Table 6:** Primer sequences, linkage groups, repeat motifs, florescent dyes used to label the forward primer and annealing temperatures for eight selected SSR loci used in molecular characterization of the quinoa accessions.

No._Locus ^a^	Forward Sequence (5′-3′) Reverse Sequence (5′-3′)	Linkage Group ^b^	Repeat Motif	Fluorescent Dye ^c^	Annealing Temp. (°C)
1_QAAT022	TGGTCGATATAGATGAACCAAA GGAGCCCAGATTGTATCTCA	23	(TTA)_29_	CFR590	52
2_QAAT024	ACCATAACAGCACCCACCTT AGGGATCAATCTTGTTCATTCA	15	(ATT)_10_	FAM	52
3_QAAT050	GGCACGTGCTGCTACTCATA ATGGCGAATGGTTAATTTGC	13	(ATT)_17_	FAM	52
4_QAAT070	TGAACAGGATCGTCATAGTCAA CGTTCATCATCTGACCCAAT	ND	(ATT)_15_	CFG540	50
5_QAAT076	GCTTCATGTGTTATAAAATGCCAAT TCTCGGCTTCCCACTAATTT	ND	(ATT)_30_	FAM	50
6_QAAT097	AAATCATTTGACTTTGTAGGTTT GATGTGATAAGGAATAATCCAA	1	(AAT)_18_	Q570	50
7_QAAT100	GGCATCCAGAGGTCAGTCTT GCAATTCTTCCTAATAACAACAACAA	10	(ACTACC)_8_ACTGTT(ATTGTT)_23_ (ATT)_5_GTT(ATT)_2_(ACT)_11_	FAM	50
8_QAAT106	TCAGTAAGATAATACCCATCAGTAAG AAAATCCCCTCTATAATTACCAA	28	(TAA)_9_	FAM	52

^a^ SSR loci described by Mason et al. [28]; ^b^ linkage groups reported by Jarvis et al. [29], ND: no data available; ^c^ CFR590: CAL Fluor Red 590; CFG 540: CAL Fluor Gold 540; Q570: Quasar 570 (LGC Biosearch Tech., Petaluma, CA, USA).

## Data Availability

The original contributions presented in this study are included in the article/Supplementary Materials. Further inquiries can be directed to the corresponding author.

## Supplementary Materials

The following supporting information can be downloaded at https://www.mdpi.com/article/10.3390/plants14050635/s1, Table S1: Allelic combinations or genotypes (G1 to G1055) found in the 268 quinoa accessions analyzed with eight microsatellite loci. The accessions are identified by the INIAP Germplasm Bank (BG) code and the analysis code used in this study (Q1 to Q268). The collection and province of origin are indicated, as well as the number of analyzed samples. The genotypes found in the improved INIAP–Tunkahuan variety are listed at the end of the table. Table S2. Allele sizes (A; in base pairs) and their frequencies (F) found for each of the eight microsatellite loci analyzed in 1067 quinoa samples. Alleles with frequencies > 0.3 are shown in bold. Table S3. Genotypes (g) and their frequencies (F) found for each of the eight microsatellite loci analyzed in 1067 quinoa samples. Genotypes with frequencies > 0.2 are shown in bold. Table S4. Analysis of molecular variance (AMOVA) of 1067 quinoa samples distributed among the three provinces (Chimborazo, Cotopaxi and Imbabura) and for the two collections (A and B). The analysis has been carried out considering both collections and also for each collection separately. Table S5. Analysis of molecular variance (AMOVA) of the 1006 quinoa samples distributed in the two clusters (390 from Cluster 1 and 616 from Cluster 2) defined by the STRUCTURE analysis. Figure S1. Genetic structure assignment model of 1067 quinoa samples. (a) Evanno’s method [60] with an optimal model of K = 2 genetic groups/clusters. (b) Grouping of Ecuadorian quinoa accession genotypes (Clusters 1 and 2). Vertical bars represent individual samples and the colors of the bars indicates the probability (membership coefficients) that a sample will be assigned to one of the identified groups (Cluster 1 in red and Cluster 2 in green).
