# Supplementary material for: Assessment of Genetic Diversity in Quinoa Landraces Cultivated in the Ecuadorian Highlands Since the Early 1980s
Source: Plants (Basel). 2025 Feb 20;14(5):635. doi: 10.3390/plants14050635 (PMC11902158; doi:10.3390/plants14050635)

Supplementary Table S1. Allelic combinations or genotypes (G1 to G1055) found in the 268 quinoa accessions analyzed with eight microsatellite loci. The accessions are identified by the INIAP Germplasm Bank (BG) code and the analysis code used in this study (Q1 to Q268). The collection and province of origin are indicated, as well as the number of analyzed samples. The genotypes found in the improved INIAP-Tunkahuan variety are listed at the end of the table.

**INIAP GB Code ***	**Analysis Code**	**Province Collection**	**No.**	**1_QAAT022**		**2_QAAT024**		**3_QAAT050**		**4_QAAT070**		**5_QAAT076**		**6_QAAT097**		**7_QAAT100**		**8_QAAT106**		**G**
ECU-254	Q1	Chimborazo Collection A	1	176	176	221	221	223	223	188	188	160	148	184	184	384	384	295	295	G1
			2	182	173	209	209	211	211	188	185	160	160	193	193	408	366	295	295	G2
			3	176	176	221	221	223	223	188	161	160	148	208	208	384	366	295	295	G3
ECU-440	Q2		4	182	182	197	197	226	226	185	185	160	160	181	181	378	366	295	295	G4
			5	185	182	221	221	226	223	188	188	160	160	205	181	378	366	295	295	G5
			6	182	182	197	197	226	226	185	185	160	160	181	181	378	378	295	295	G6
			7	182	182	197	197	226	226	185	185	160	160	181	181	366	366	295	295	G7
ECU-442	Q3		8	182	182	197	197	223	223	161	161	148	148	181	181	415	415	295	295	G8
			9	176	176	221	221	226	226	188	188	160	157	181	181	396	396	295	292	G9
			10	182	182	197	197	232	232	161	161	148	148	181	181	421	421	295	295	G10
			11	182	176	221	221	223	223	188	188	160	157	208	181	396	396	295	292	G11
ECU-443	Q4		12	179	179	200	200	220	220	161	161	148	148	208	208	421	421	295	295	G12
			13	199	176	200	200	217	217	161	161	148	148	208	208	366	366	295	295	G13
			14	182	182	209	209	223	223	161	161	154	154	208	184	396	396	295	295	G14
			15	199	179	200	200	207	207	161	161	148	148	211	208	421	421	295	295	G15
ECU-444	Q5		16	182	182	221	221	226	226	161	161	160	160	205	205	421	421	295	295	G16
			17	182	182	221	209	226	226	161	161	160	160	181	181	408	408	304	301	G17
			18	182	182	221	221	195	195	161	161	160	160	181	181	408	408	295	295	G18
			19	182	173	221	209	226	223	188	161	160	160	205	181	408	366	304	301	G19
ECU-445	Q6		20	182	182	245	245	198	198	182	173	160	160	187	184	384	357	301	301	G20
			21	182	182	245	245	195	195	167	167	160	160	181	181	384	384	301	301	G21
			22	173	173	218	200	226	226	185	185	160	160	211	211	384	384	295	295	G22
			23	196	182	218	200	226	211	173	161	160	160	211	196	421	402	295	295	G23
ECU-487	Q7		24	185	182	224	197	226	226	161	161	154	148	181	181	408	408	301	317	G24
			25	193	193	224	197	229	229	161	161	154	148	181	181	408	408	301	292	G25
			26	193	193	224	197	226	226	161	161	154	148	181	181	408	408	301	292	G26
			27	202	193	224	197	226	226	161	161	154	148	208	181	408	366	301	292	G27
ECU-488	Q8		28	179	179	224	224	226	226	161	161	154	154	208	208	396	396	295	295	G28
			29	179	179	224	224	220	220	161	161	154	154	208	208	408	408	292	292	G29
ECU-493	Q9		30	182	182	197	197	226	226	185	185	148	148	181	181	366	366	301	301	G30
			31	182	182	197	197	223	223	185	161	148	148	211	211	421	421	301	301	G31
			32	182	182	197	197	223	223	161	161	148	148	211	211	366	366	301	301	G32

Supplementary Table S1. Continued.

INIAP GB Code *	Analysis Code	Province Collection	No.	1_QAAT022		2_QAAT024		3_QAAT050		4_QAAT070		5_QAAT076		6_QAAT097		7_QAAT100		8_QAAT106		G
			33	196	196	221	221	195	195	173	173	160	160	208	208	402	402	295	295	G33
			34	185	173	221	221	226	223	173	161	160	160	205	181	378	366	295	295	G34
			35	196	196	197	197	226	226	173	173	160	160	181	181	408	408	295	295	G35
ECU-495	Q10		36	196	196	197	197	226	226	173	173	160	160	211	211	402	402	295	295	G36
			37	199	199	197	197	226	226	173	173	160	160	208	208	402	402	295	295	G37
			38	182	182	209	209	223	223	188	188	160	160	193	193	357	357	301	301	G38
			39	193	182	218	218	223	195	188	188	163	160	208	190	378	357	301	301	G39
ECU-541	Q11		40	205	205	218	218	198	198	188	188	163	160	208	196	396	396	301	301	G40
			41	196	196	209	209	223	223	188	188	160	160	181	181	384	384	301	301	G41
			42	182	182	218	218	223	223	188	188	163	160	193	181	357	357	301	301	G42
			43	179	179	197	197	195	195	161	161	148	148	208	208	396	396	295	295	G43
ECU-609	Q12		44	179	179	197	197	195	195	161	161	148	148	181	181	396	396	295	295	G44
			45	196	196	197	197	223	223	188	188	145	145	208	208	384	384	295	295	G45
			46	196	196	197	197	223	195	161	161	148	148	208	181	396	396	295	295	G46
			47	179	179	209	209	195	195	161	161	160	160	208	208	402	402	295	295	G47
			48	182	182	215	215	211	211	173	173	160	160	181	181	396	396	295	295	G48
ECU-610	Q13		49	182	182	215	215	211	211	173	173	160	160	208	208	421	421	295	295	G49
		Chimborazo Collection A	50	235	196	209	209	220	195	161	161	160	160	184	184	402	366	295	295	G50
			51	182	182	215	215	211	211	173	173	160	160	208	208	421	396	295	295	G51
ECU-611	Q14		52	179	179	197	197	195	195	161	161	148	148	208	208	396	396	295	295	G43
			53	179	179	197	197	195	195	161	161	148	145	208	208	396	396	295	295	G52
ECU-612	Q15		54	179	179	197	197	195	195	161	161	148	145	181	181	396	396	295	295	G53
			55	179	179	197	197	223	195	161	161	148	145	208	208	396	384	295	295	G54
			56	196	193	197	197	195	195	161	161	148	148	208	208	396	396	295	295	G55
			57	196	196	224	224	195	195	161	161	160	160	181	181	408	408	295	295	G56
ECU-613	Q16		58	196	196	221	221	195	195	161	161	160	160	181	181	384	384	295	295	G57
			59	196	196	224	224	195	195	161	161	160	160	181	181	384	384	295	295	G58
			60	196	196	221	221	226	226	161	161	160	160	208	208	402	366	295	295	G59
ECU-615	Q17		61	196	196	197	197	195	195	188	188	145	145	208	208	402	402	295	295	G60
			62	196	196	197	197	195	195	188	161	145	145	208	208	402	402	295	295	G61
			63	188	188	221	221	214	214	185	161	148	148	208	208	372	372	295	295	G62
ECU-622	Q18		64	188	188	221	221	214	214	185	185	148	148	208	208	372	372	295	295	G63
			65	185	185	221	221	214	214	185	185	148	148	208	208	372	372	295	295	G64
ECU-623	Q19		66	176	176	215	215	195	195	182	182	148	148	208	208	366	366	298	298	G65

Supplementary Table S1. Continued.

INIAP GB Code *	Analysis Code	Province Collection	No.	1_QAAT022		2_QAAT024		3_QAAT050		4_QAAT070		5_QAAT076		6_QAAT097		7_QAAT100		8_QAAT106		G
		Chimborazo Collection A	67	176	176	215	197	226	195	185	182	160	148	208	208	354	354	301	298	G66
			68	176	176	215	215	195	195	182	182	148	148	208	208	354	354	298	298	G67
			69	176	176	215	215	195	195	182	182	148	148	205	205	354	354	298	298	G68
ECU-632	Q20		70	173	173	221	221	195	195	161	161	148	148	211	211	381	381	295	295	G69
			71	179	179	197	197	232	232	173	173	160	160	181	181	390	390	298	298	G70
			72	182	182	221	221	223	223	161	161	148	148	181	181	384	384	295	295	G71
			73	179	179	197	197	232	232	161	161	160	160	181	181	390	390	298	298	G72
ECU-232	Q21	Cotopaxi Collection A	74	179	179	215	215	211	211	188	188	157	157	211	211	396	366	317	313	G73
			75	179	179	200	200	226	226	182	182	157	157	208	208	366	366	301	301	G74
			76	185	185	200	200	211	211	182	182	157	157	208	208	366	366	301	301	G75
			77	185	185	215	215	226	195	188	188	157	157	211	211	372	372	317	313	G76
ECU-328	Q22		78	205	205	197	197	217	195	188	188	151	151	205	205	366	366	295	295	G77
			79	202	202	218	218	211	211	161	161	148	148	208	208	366	366	298	298	G78
			80	205	205	197	197	195	195	188	188	151	151	187	187	366	366	295	295	G79
ECU-341	Q23		81	199	199	197	197	214	214	173	173	148	148	181	181	366	366	298	298	G80
			82	202	202	197	197	204	204	173	173	148	148	181	181	366	366	298	298	G81
			83	199	199	197	197	204	204	188	188	148	148	181	181	366	366	298	298	G82
			84	196	196	221	221	211	211	188	188	160	160	205	205	384	384	295	295	G83
ECU-347	Q24		85	199	196	221	221	220	220	182	182	163	160	208	187	378	366	301	292	G84
			86	196	196	221	215	232	232	188	188	160	157	208	208	378	366	317	317	G85
			87	196	196	221	215	220	220	188	188	160	157	187	187	378	378	317	317	G86
ECU-352	Q25		88	199	199	221	215	226	226	188	188	148	145	190	190	390	390	295	292	G87
			89	182	182	224	221	226	226	188	188	148	148	208	208	372	372	295	295	G88
			90	179	179	221	215	226	211	182	182	148	145	190	190	390	390	295	292	G89
			91	199	182	224	221	229	226	188	161	148	148	208	190	372	366	295	295	G90
			92	199	196	221	215	226	226	188	188	148	145	214	190	396	390	295	292	G91
ECU-362	Q26		93	182	182	221	200	195	195	185	185	160	151	181	181	366	366	301	301	G92
			94	196	196	218	218	232	232	188	188	157	157	208	208	402	402	295	295	G93
			95	182	182	221	200	192	192	185	185	160	151	181	181	366	366	301	301	G94
			96	235	182	221	200	195	195	185	161	160	151	211	181	366	357	301	301	G95
ECU-424	Q27		97	182	182	197	197	226	195	161	161	148	148	187	187	396	366	317	317	G96
			98	182	182	197	197	195	195	191	191	148	148	187	187	396	366	317	317	G97
			99	185	182	197	197	207	195	191	188	148	148	208	187	396	366	317	317	G98
			100	188	188	224	221	226	217	188	188	160	151	187	187	396	366	292	292	G99

Supplementary Table S1. Continued.

INIAP GB Code *	Analysis Code	Province Collection	No.	1_QAAT022		2_QAAT024		3_QAAT050		4_QAAT070		5_QAAT076		6_QAAT097		7_QAAT100		8_QAAT106		G
ECU-426	Q28	Cotopaxi Collection A	101	185	185	215	215	207	207	188	188	151	151	190	190	384	384	295	295	G100
			102	199	199	224	224	226	226	188	161	148	148	211	211	381	381	295	295	G101
			103	199	199	224	224	226	226	188	161	148	148	211	211	372	372	295	295	G102
			104	182	182	224	224	226	226	188	188	148	148	208	208	415	415	295	295	G103
ECU-427	Q29		105	205	205	221	221	226	226	182	161	160	160	208	208	402	402	317	317	G104
			106	182	182	221	221	198	198	188	182	160	160	211	211	396	390	317	317	G105
			107	179	179	221	221	198	198	182	182	160	160	211	211	390	390	317	317	G106
ECU-430	Q30		108	199	176	218	197	211	195	188	161	157	148	211	181	366	366	317	317	G107
			109	199	199	218	197	211	211	188	188	157	148	211	211	366	366	317	317	G108
			110	199	199	218	197	214	214	188	188	157	148	208	208	372	372	317	317	G109
			111	199	173	218	197	226	214	188	185	157	148	211	181	366	366	317	317	G110
			112	182	179	200	197	220	220	188	173	160	160	211	211	408	402	295	295	G111
ECU-431	Q31		113	176	176	197	197	229	229	188	188	160	160	205	205	396	396	295	295	G112
			114	179	179	227	227	226	226	182	161	148	148	208	208	390	390	320	320	G113
			115	179	176	227	227	229	226	188	161	148	148	208	205	396	390	320	320	G114
			116	179	176	197	197	229	229	188	188	160	160	205	205	396	396	295	295	G115
ECU-450	Q32		117	182	182	221	197	226	226	197	197	148	148	211	211	366	366	320	295	G116
			118	179	179	221	197	214	214	191	188	148	148	208	208	402	366	320	295	G117
			119	205	182	227	197	232	211	188	188	160	148	208	208	396	366	313	295	G118
			120	205	205	227	197	232	232	188	188	160	148	208	208	396	396	313	295	G119
ECU-452	Q33		121	176	176	215	197	226	226	188	188	148	148	208	208	366	366	292	292	G120
			122	176	176	215	215	211	211	188	188	148	148	208	208	366	366	317	317	G121
ECU-479	Q34		123	182	182	197	197	207	207	185	185	148	148	208	208	366	366	317	301	G122
			124	182	182	197	197	207	207	185	185	148	148	208	208	402	402	301	301	G123
			125	182	182	197	197	207	207	191	191	148	148	208	208	408	408	301	301	G124
			126	182	182	197	197	220	220	191	191	148	148	208	208	366	366	317	301	G125
			127	182	182	197	197	207	207	191	185	148	148	208	208	366	366	317	301	G126
ECU-525	Q35		128	188	188	197	197	198	198	161	161	157	157	181	181	415	415	313	313	G127
			129	185	185	197	197	198	198	161	161	157	157	181	181	415	415	313	313	G128
			130	188	188	197	197	198	198	188	161	148	148	208	181	415	415	295	295	G129
ECU-528	Q36		131	179	179	236	197	195	195	188	188	172	157	196	181	366	366	292	292	G130
			132	179	179	209	209	217	204	191	191	172	172	196	196	381	381	317	317	G131
			133	179	179	236	197	226	198	188	161	172	157	187	187	354	354	292	292	G132
			134	179	179	236	197	198	198	185	185	172	157	187	181	408	408	292	292	G133

Supplementary Table S1. Continued.

INIAP GB Code *	Analysis Code	Province Collection	No.	1_QAAT022		2_QAAT024		3_QAAT050		4_QAAT070		5_QAAT076		6_QAAT097		7_QAAT100		8_QAAT106		G
		Cotopaxi Collection A	135	179	179	209	209	214	204	185	170	172	172	196	196	381	366	317	317	G134
ECU-529	Q37		136	182	179	209	197	201	198	188	188	169	151	199	196	411	411	317	307	G135
			137	182	179	212	212	204	204	185	185	169	169	196	187	381	354	317	317	G136
			138	182	179	212	212	226	204	191	191	169	169	187	181	381	381	317	317	G137
			139	182	179	209	197	195	195	188	185	169	151	193	193	408	366	317	307	G138
			140	182	179	209	197	220	204	188	188	169	151	205	196	366	357	317	307	G139
ECU-617	Q38		141	196	179	215	215	211	195	188	188	148	148	175	175	366	366	295	295	G140
			142	199	179	197	197	211	211	188	188	148	148	208	208	372	372	295	295	G141
			143	199	179	197	197	207	207	188	188	148	148	211	211	366	366	295	295	G142
			144	210	179	197	197	211	211	188	188	148	148	175	175	366	366	295	295	G143
			145	199	179	215	215	211	211	188	188	148	148	208	208	366	366	295	295	G144
ECU-2323	Q39		146	179	179	221	221	207	207	188	188	157	157	205	205	366	366	295	295	G145
			147	199	179	221	221	207	207	188	188	148	148	190	190	372	372	317	317	G146
			148	199	179	221	221	207	207	188	188	148	148	208	208	372	372	317	317	G147
ECU-2324	Q40		149	196	179	218	197	207	207	188	188	148	148	208	208	366	366	317	317	G148
			150	196	179	218	197	195	195	188	188	148	148	208	208	372	366	317	317	G149
			151	179	179	218	197	211	211	188	188	148	148	211	211	366	366	317	317	G150
			152	196	179	221	221	207	207	188	188	148	148	208	208	372	372	317	317	G151
ECU-234	Q41	Imbabura Collection A	153	179	179	197	197	226	198	182	182	160	154	211	181	366	366	295	295	G152
			154	179	179	221	197	226	226	185	185	160	154	208	208	408	408	295	295	G153
			155	179	179	221	197	223	195	191	191	160	154	208	208	390	390	295	295	G154
			156	179	179	221	197	195	195	185	185	160	154	193	193	366	366	295	295	G155
			157	179	176	197	197	226	195	188	185	160	154	211	181	366	366	295	295	G156
ECU-321	Q42		158	185	185	224	194	226	226	185	185	154	148	208	208	384	384	301	301	G157
			159	182	182	194	194	226	226	185	185	154	154	208	208	396	396	295	295	G158
			160	196	196	224	194	238	238	185	185	154	148	178	178	384	384	301	301	G159
			161	182	182	194	194	226	220	185	173	154	154	208	178	396	396	295	295	G160
			162	199	185	224	194	238	198	185	176	154	148	208	208	384	354	301	301	G161
ECU-353	Q43		163	179	179	221	221	198	198	188	161	160	160	181	181	396	396	295	295	G162
			164	182	182	197	197	198	198	188	188	148	148	181	181	396	396	301	295	G163
			165	182	182	197	197	198	198	188	188	148	148	181	181	378	378	301	295	G164
			166	182	182	197	197	223	195	191	161	148	148	208	208	384	366	301	295	G165
ECU-354	Q44		167	179	179	215	215	198	198	188	188	160	160	181	181	396	396	295	295	G166
ECU-358	Q45		168	182	179	215	209	226	195	173	161	160	160	208	181	396	396	304	301	G167

Supplementary Table S1. Continued.

INIAP GB Code *	Analysis Code	Province Collection	No.	1_QAAT022		2_QAAT024		3_QAAT050		4_QAAT070		5_QAAT076		6_QAAT097		7_QAAT100		8_QAAT106		G
		Imbabura Collection A	169	182	182	215	215	198	198	170	170	160	160	211	193	396	357	295	295	G168
			170	182	182	215	215	198	198	188	188	160	160	211	193	396	396	295	295	G169
			171	182	182	215	215	201	195	188	182	160	160	211	181	402	396	295	295	G170
			172	182	182	215	215	198	198	188	188	160	160	211	211	396	372	295	295	G171
ECU-361	Q46		173	176	176	215	197	198	198	188	188	163	160	181	181	396	396	301	301	G172
			174	176	176	215	215	195	195	188	188	160	160	181	181	396	396	295	295	G173
			175	176	176	215	197	198	195	188	161	163	160	181	181	396	372	301	301	G174
ECU-374	Q47		176	202	196	215	215	226	226	188	188	160	145	208	205	396	366	317	313	G175
			177	205	199	215	215	232	207	188	188	145	145	208	181	396	366	317	317	G176
			178	205	205	215	215	232	232	188	188	145	145	181	181	396	396	317	317	G177
			179	205	205	215	215	232	232	188	188	145	145	208	208	366	366	317	317	G178
ECU-375	Q48		180	196	196	218	215	232	232	188	188	160	160	208	208	384	384	295	295	G179
			181	199	199	218	215	232	226	188	188	160	160	208	208	366	366	295	295	G180
			182	199	199	221	221	232	226	188	188	160	160	208	208	366	366	295	295	G181
			183	199	199	221	221	207	207	188	188	160	160	208	208	390	390	295	295	G182
			184	196	196	218	215	211	211	188	188	160	160	208	208	366	366	295	295	G183
ECU-480	Q49		185	182	182	221	221	198	198	188	188	160	160	181	181	396	396	295	295	G184
			186	182	182	221	221	220	195	188	161	160	160	208	181	396	396	295	295	G185
			187	182	182	221	221	220	220	185	185	160	160	208	208	396	396	295	295	G186
ECU-532	Q50		188	188	182	197	197	204	198	173	161	148	148	196	181	390	366	317	301	G187
			189	188	188	197	197	204	204	182	182	160	160	196	196	390	390	301	301	G188
			190	188	188	197	197	192	192	182	182	160	160	196	196	390	390	301	301	G189
			191	188	188	197	197	204	204	182	182	160	160	199	196	390	390	301	301	G190
ECU-533	Q51		192	188	188	239	239	201	201	182	182	157	157	190	190	458	354	292	292	G191
			193	219	219	239	239	198	198	182	182	157	157	190	190	458	458	292	292	G192
			194	188	188	239	239	201	201	182	182	157	157	190	190	458	458	292	292	G193
			195	219	219	239	239	198	198	182	182	157	157	187	187	458	458	292	292	G194
ECU-547	Q52		196	202	202	215	215	211	211	188	188	145	145	181	181	366	366	317	317	G195
			197	202	202	215	215	211	207	188	188	145	145	208	181	390	372	317	317	G196
			198	199	199	221	197	195	195	191	188	148	148	181	181	372	354	317	317	G197
ECU-594	Q53		199	182	182	197	197	195	195	185	185	160	160	208	181	415	396	329	329	G198
			200	182	182	197	197	195	195	185	185	160	160	208	208	366	366	329	329	G199
			201	182	182	197	197	195	195	191	185	160	160	208	208	366	366	329	329	G200
			202	182	182	197	197	211	195	185	185	160	160	181	181	415	415	301	301	G201

Supplementary Table S1. Continued.

INIAP GB Code *	Analysis Code	Province Collection	No.	1_QAAT022		2_QAAT024		3_QAAT050		4_QAAT070		5_QAAT076		6_QAAT097		7_QAAT100		8_QAAT106		G
ECU-595	Q54	Imbabura Collection A	203	182	182	221	221	226	217	191	185	160	148	217	217	366	366	301	301	G202
			204	182	182	221	221	226	226	191	188	160	148	214	214	396	396	301	301	G203
			205	182	182	221	221	195	195	185	161	160	148	208	181	366	366	301	301	G204
			206	185	185	221	197	217	217	191	185	160	148	208	208	396	396	301	301	G205
			207	185	185	221	197	226	226	191	185	160	148	208	208	396	396	301	301	G206
ECU-596	Q55		208	182	182	221	197	195	195	191	191	160	148	208	208	366	366	301	301	G207
			209	182	179	221	197	195	195	188	188	160	148	181	181	372	372	301	301	G208
ECU-597	Q56		210	182	179	197	197	195	195	185	185	151	148	208	208	366	366	295	295	G209
			211	182	182	197	197	195	195	185	185	148	148	208	208	366	366	295	295	G210
ECU-598	Q57		212	182	182	218	218	226	195	188	185	148	148	205	181	396	366	301	301	G211
			213	182	179	218	197	195	195	188	185	157	148	181	181	366	366	301	301	G212
ECU-599	Q58		214	185	182	197	197	223	195	191	185	163	154	211	208	402	378	295	295	G213
			215	182	179	221	221	226	195	185	182	148	148	208	208	366	366	301	301	G214
			216	182	182	197	197	195	195	191	191	163	154	208	208	402	396	295	295	G215
			217	182	182	221	221	198	198	185	185	148	148	181	181	366	366	301	301	G216
ECU-600	Q59		218	188	182	215	197	226	195	185	182	157	157	208	208	366	366	301	301	G217
			219	182	182	215	197	195	195	185	182	157	157	208	208	366	366	301	301	G218
			220	182	182	215	197	195	195	182	182	157	157	208	208	366	366	301	301	G219
			221	182	182	221	197	226	226	185	185	157	157	208	208	366	366	301	301	G220
			222	182	182	221	197	226	195	188	188	157	157	208	208	366	366	301	301	G221
ECU-636	Q60		223	199	199	221	200	226	226	188	188	157	157	208	208	372	372	301	301	G222
			224	199	199	218	200	211	211	188	188	157	157	208	208	384	384	301	301	G223
			225	199	199	221	200	211	211	182	182	157	157	208	208	372	372	301	301	G224
ECU-640	Q61		226	185	182	221	197	226	223	185	185	157	148	211	208	366	354	301	295	G225
			227	182	182	221	197	195	195	185	161	157	148	211	211	396	366	301	295	G226
			228	182	182	221	197	195	195	185	185	157	148	208	208	366	366	301	295	G227
			229	196	196	221	221	223	223	161	161	145	145	211	211	427	427	304	304	G228
ECU-27192	Q62	Chimborazo Collection B	230	196	182	218	200	223	195	161	161	160	160	211	208	421	408	304	295	G229
			231	179	179	218	200	226	195	185	185	160	160	211	181	421	421	304	295	G230
			232	182	173	221	200	220	195	161	161	160	157	211	205	384	384	298	295	G231
			233	176	176	221	200	223	223	161	161	160	157	181	181	366	366	298	295	G232
			234	182	182	218	200	195	195	161	161	160	160	208	208	421	396	304	295	G233
HDEN-2	Q63		235	179	173	221	197	223	220	161	161	160	160	208	208	440	421	304	301	G234
			236	182	173	221	200	223	207	173	161	160	163	208	181	427	372	301	295	G235

Supplementary Table S1. Continued.

INIAP GB Code *	Analysis Code	Province Collection	No.	1_QAAT022		2_QAAT024		3_QAAT050		4_QAAT070		5_QAAT076		6_QAAT097		7_QAAT100		8_QAAT106		G
		Chimborazo Collection B	237	182	182	221	200	220	195	173	161	160	163	208	181	421	384	301	295	G236
			238	182	173	221	197	223	207	161	161	160	160	181	181	427	372	304	301	G237
			239	182	182	221	200	226	226	161	161	160	163	205	181	384	384	301	295	G238
ECU-27193	Q64		240	176	176	221	221	223	223	188	188	148	148	184	184	384	384	295	295	G239
			241	176	176	221	221	223	223	161	161	148	148	208	208	366	366	295	295	G240
			242	185	185	221	197	192	192	194	194	148	148	211	181	396	396	295	295	G241
ECU-27194	Q65		243	185	185	221	221	195	195	194	194	148	148	181	181	366	366	295	295	G242
			244	182	182	221	200	195	195	194	194	160	160	208	208	372	372	298	295	G243
			245	182	182	221	221	226	226	194	194	148	148	211	211	396	396	295	295	G244
			246	185	185	221	200	207	195	194	194	160	160	208	181	366	366	298	295	G245
			247	182	173	221	200	195	195	161	161	160	160	208	181	396	387	298	295	G246
ECU-27195	Q66		248	188	185	221	197	198	195	161	161	160	160	217	184	396	396	301	301	G247
			249	185	185	221	197	195	195	161	161	160	160	208	181	396	387	301	301	G248
			250	182	182	221	197	229	226	188	173	160	145	184	181	427	421	298	295	G249
			251	185	182	221	197	229	220	161	161	160	160	208	184	396	384	301	301	G250
			252	182	182	221	197	226	195	161	161	160	160	208	184	446	384	301	301	G251
ECU-27196	Q67		253	182	182	221	200	223	195	161	161	163	160	184	184	421	366	295	295	G252
			254	182	182	221	200	220	195	173	161	163	160	208	184	421	402	295	295	G253
			255	182	173	221	200	223	195	173	161	163	160	205	184	421	384	295	295	G254
			256	182	176	221	200	226	195	188	185	163	160	208	184	421	396	295	295	G255
			257	182	182	221	221	195	195	161	161	160	160	208	193	396	396	317	317	G256
ECU-27198	Q68		258	185	182	197	197	226	195	161	161	160	160	208	181	366	366	292	292	G257
			259	182	179	197	197	226	195	173	161	160	160	208	181	381	366	292	292	G258
			260	182	179	221	221	220	220	161	161	160	160	211	181	421	384	301	301	G259
			261	182	182	197	197	226	220	173	161	160	160	208	181	408	366	292	292	G260
ECU-27199	Q69		262	182	182	197	197	226	226	173	173	160	160	208	208	408	408	292	292	G261
			263	182	182	197	197	226	195	161	161	160	160	208	184	446	384	292	292	G262
			264	179	179	197	197	223	195	161	161	160	160	208	208	433	366	298	295	G263
			265	179	179	197	197	226	226	161	161	160	160	208	208	384	384	298	295	G264
			266	179	173	197	197	195	195	161	161	160	160	208	208	366	366	298	295	G265
ECU-27200	Q70		267	193	173	200	197	229	195	161	161	160	160	205	205	421	384	295	295	G266
			268	196	179	200	197	223	195	188	161	160	160	211	181	402	366	295	295	G267
			269	196	182	200	197	232	220	161	161	160	160	211	181	408	384	295	295	G268
			270	182	173	200	197	223	195	161	161	160	160	205	205	421	421	295	295	G269

Supplementary Table S1. Continued.

INIAP GB Code *	Analysis Code	Province Collection	No.	1_QAAT022		2_QAAT024		3_QAAT050		4_QAAT070		5_QAAT076		6_QAAT097		7_QAAT100		8_QAAT106		G
ECU-27201	Q71	Chimborazo Collection B	271	196	196	197	197	220	195	173	161	163	160	208	181	402	366	295	295	G270
			272	196	182	197	197	226	220	188	161	163	160	211	181	402	384	295	295	G271
			273	182	182	197	197	195	195	188	188	163	160	211	211	402	402	295	295	G272
			274	199	196	197	197	226	195	161	161	160	160	211	181	427	427	301	295	G273
			275	199	193	197	197	226	195	161	161	160	160	187	181	440	427	301	295	G274
ECU-27202	Q72		276	182	182	221	209	223	223	185	185	160	160	181	181	384	384	295	295	G275
			277	179	179	197	197	232	232	173	173	160	160	181	181	390	390	298	298	G70
ECU-27203	Q73		278	182	182	200	197	220	220	161	161	160	160	211	211	366	366	295	295	G276
			279	182	182	197	197	195	195	161	161	142	142	214	214	408	408	295	295	G277
			280	179	179	200	197	195	195	161	161	160	160	181	181	366	366	295	295	G278
			281	182	182	200	197	195	195	161	161	160	160	208	208	366	366	295	295	G279
ECU-27204	Q74		282	182	182	221	221	229	229	188	188	160	160	190	190	402	402	295	295	G280
			283	182	182	221	221	229	229	161	161	160	160	190	190	402	402	295	295	G281
			284	182	182	221	197	229	211	194	188	160	160	190	181	402	402	295	292	G282
ECU-27205	Q75		285	193	193	209	197	226	195	173	173	160	148	181	181	396	378	295	292	G283
			286	179	179	197	197	223	195	173	161	148	148	181	181	402	384	304	295	G284
			287	199	193	209	197	226	195	173	173	160	148	211	208	396	384	295	292	G285
			288	199	196	197	197	223	223	173	173	148	148	181	181	402	378	304	295	G286
			289	196	193	209	197	226	195	173	173	160	148	211	211	384	384	295	292	G287
ECU-27206	Q76		290	193	193	221	221	226	226	161	161	160	148	211	181	402	402	295	295	G288
			291	193	179	197	197	223	195	185	173	160	148	184	181	396	366	304	304	G289
			292	182	179	197	197	223	195	173	161	160	148	208	193	421	378	304	304	G290
			293	182	182	221	221	223	195	161	161	160	148	211	208	366	366	295	295	G291
			294	193	182	221	221	226	220	188	161	160	148	211	184	402	366	295	295	G292
ECU-27208	Q77		295	179	179	209	209	195	195	161	161	160	148	208	208	402	402	295	295	G293
			296	182	182	215	215	211	211	173	173	160	148	208	208	421	421	295	295	G294
			297	196	196	209	209	220	195	161	161	160	148	184	184	402	366	295	295	G295
			298	182	182	215	215	211	211	173	173	160	148	208	208	421	396	295	295	G296
ECU-27209	Q78		299	182	179	221	197	226	223	161	161	160	157	196	181	396	396	295	295	G297
			300	182	176	221	215	226	223	161	161	160	160	211	181	396	384	295	295	G298
			301	176	173	221	197	223	195	173	161	160	157	181	181	427	396	295	295	G299
ECU-27210	Q79		302	185	173	221	197	226	220	161	161	157	157	181	181	421	372	295	292	G300
			303	185	182	221	197	195	195	161	161	160	160	208	181	440	421	304	295	G301
			304	196	179	221	197	220	195	161	161	160	160	208	181	421	421	304	295	G302

Supplementary Table S1. Continued.

INIAP GB Code *	Analysis Code	Province Collection	No.	1_QAAT022		2_QAAT024		3_QAAT050		4_QAAT070		5_QAAT076		6_QAAT097		7_QAAT100		8_QAAT106		G
ECU-27211	Q80	Chimborazo Collection B	305	179	179	221	197	195	195	173	161	160	160	208	208	433	433	298	295	G303
			306	179	179	221	197	195	195	173	161	160	160	208	208	433	421	298	295	G304
			307	182	179	221	197	232	195	185	161	163	160	211	205	427	372	298	295	G305
ECU-27212	Q81		308	182	182	197	197	226	195	161	161	160	160	184	181	402	396	292	292	G306
			309	182	182	197	197	195	195	161	161	160	160	181	181	421	372	292	292	G307
			310	182	182	197	197	217	217	161	161	160	160	202	202	421	372	292	292	G308
			311	182	182	197	197	220	217	161	161	160	160	202	184	372	372	292	292	G309
			312	182	179	221	200	226	226	173	161	163	160	184	184	421	396	295	295	G310
ECU-27214	Q82		313	182	182	221	197	217	217	161	161	160	160	202	202	421	372	292	292	G311
			314	182	182	221	197	223	223	161	161	145	145	208	208	421	421	295	292	G312
			315	182	182	221	197	217	217	161	161	160	160	181	181	372	372	292	292	G313
			316	182	179	221	197	226	217	161	161	145	145	208	202	421	421	295	292	G314
ECU-27215	Q83		317	176	176	197	197	229	229	161	161	157	154	208	208	427	396	298	295	G315
			318	176	176	197	197	195	195	161	161	157	154	181	181	421	396	298	295	G316
			319	182	176	197	197	229	223	161	161	157	154	208	208	427	421	298	295	G317
			320	185	185	197	197	229	229	173	173	160	160	208	208	396	396	295	295	G318
ECU-27216	Q84		321	179	179	221	197	223	195	188	161	160	160	208	181	433	421	301	301	G319
			322	176	176	224	224	229	195	161	161	160	154	208	208	427	427	301	301	G320
			323	179	179	221	197	220	195	188	161	160	160	184	181	384	366	301	301	G321
			324	179	179	221	197	226	220	188	161	160	160	184	181	366	366	301	301	G322
ECU-27218	Q85		325	182	182	197	197	211	211	185	185	148	148	208	181	366	366	295	295	G323
			326	182	182	197	197	195	195	161	161	142	142	214	214	408	366	295	295	G324
			327	179	179	197	197	195	195	161	161	148	148	181	181	366	366	295	295	G325
			328	182	182	197	197	220	220	185	161	148	148	211	211	366	366	295	295	G326
			329	182	182	197	197	195	195	161	161	148	148	208	208	366	366	295	295	G327
ECU-27219	Q86		330	173	173	221	197	195	195	161	161	148	148	202	181	378	366	295	295	G328
			331	182	176	221	197	226	226	188	173	160	148	202	181	378	378	295	295	G329
			332	176	176	221	197	195	195	188	161	148	148	202	181	378	378	295	295	G330
ECU-27221	Q87		333	193	193	197	197	226	226	161	161	160	160	211	211	402	402	304	304	G331
			334	193	179	197	197	223	195	185	173	160	160	184	181	396	366	304	304	G332
			335	182	182	197	197	223	195	161	161	148	148	211	208	366	366	317	317	G333
			336	199	199	197	197	214	214	188	188	148	148	211	211	366	366	317	317	G334
ECU-27221	Q88		337	196	182	200	197	226	220	188	161	160	148	211	181	402	384	295	295	G335
			338	196	196	197	197	220	195	173	161	160	148	208	181	402	366	295	295	G336

Supplementary Table S1. Continued.

INIAP GB Code *	Analysis Code	Province Collection	No.	1_QAAT022		2_QAAT024		3_QAAT050		4_QAAT070		5_QAAT076		6_QAAT097		7_QAAT100		8_QAAT106		G
		Chimborazo Collection B	339	182	179	197	197	223	195	173	161	160	148	208	193	421	378	295	295	G337
			340	193	182	200	197	226	220	188	161	160	148	211	184	402	366	295	295	G338
			341	199	196	197	197	226	195	161	161	160	148	211	181	427	427	295	295	G339
ECU-27224	Q89		342	182	182	197	197	217	195	161	161	160	148	208	181	396	390	298	295	G340
			343	182	182	221	221	226	226	161	161	160	148	208	208	366	366	298	298	G341
			344	182	182	221	221	226	226	161	161	160	148	181	181	421	354	298	298	G342
ECU-27225	Q90		345	173	173	221	197	226	226	188	188	148	145	208	181	366	366	295	295	G343
			346	182	182	221	197	217	217	161	161	148	145	181	181	366	366	295	295	G344
			347	173	173	221	197	226	226	161	161	148	145	208	181	402	402	295	295	G345
			348	173	173	221	221	226	226	188	188	157	157	181	181	402	366	298	295	G346
			349	196	196	221	221	195	195	185	185	157	157	208	181	415	415	298	295	G347
ECU-27226	Q91		350	190	185	197	197	223	223	161	161	154	154	214	214	402	402	301	301	G348
			351	202	202	197	197	229	229	161	161	154	154	208	208	402	402	301	301	G349
			352	202	202	197	197	229	229	161	161	154	154	211	208	402	402	301	301	G350
			353	190	190	221	221	229	223	161	161	160	160	181	181	421	421	298	295	G351
			354	202	190	221	221	223	195	188	161	160	160	208	181	402	366	298	295	G352
ECU-27227	Q92		355	202	202	221	221	223	195	161	161	154	154	181	181	402	402	301	295	G353
			356	202	202	197	197	223	195	188	188	160	160	208	208	421	366	301	301	G354
			357	202	202	197	197	223	223	188	188	160	160	208	208	421	421	301	301	G355
			358	202	202	197	197	223	223	188	188	160	160	208	208	421	366	301	301	G356
			359	202	202	197	197	223	223	188	161	160	160	208	208	402	402	301	301	G357
ECU-27228	Q93		360	179	179	209	209	226	195	173	161	160	160	211	208	372	366	301	301	G358
			361	193	179	197	197	195	195	161	161	145	145	208	208	421	408	298	298	G359
			362	176	176	209	209	226	195	173	161	160	160	208	208	372	372	301	301	G360
			363	179	179	197	197	195	195	161	161	145	145	208	208	408	408	298	298	G361
			364	193	179	197	197	195	195	188	161	145	145	208	208	421	408	298	298	G362
ECU-27229	Q94		365	182	182	221	221	195	195	188	185	160	145	208	208	402	366	295	295	G363
			366	179	173	221	197	220	195	182	161	160	145	211	181	408	408	298	295	G364
			367	182	182	221	221	211	195	188	185	160	145	208	208	366	366	295	295	G365
			368	179	179	221	221	195	195	188	188	160	145	208	184	402	402	295	295	G366
			369	196	179	221	221	226	195	188	188	160	145	208	208	405	405	295	295	G367
ECU-27230	Q95		370	179	179	197	197	223	223	161	161	160	145	211	184	402	396	301	295	G368
			371	196	179	197	197	195	195	173	173	160	145	181	181	408	402	301	295	G369
			372	182	176	197	197	220	195	173	173	154	154	184	184	415	415	304	295	G370

Supplementary Table S1. Continued.

INIAP GB Code *	Analysis Code	Province Collection	No.	1_QAAT022		2_QAAT024		3_QAAT050		4_QAAT070		5_QAAT076		6_QAAT097		7_QAAT100		8_QAAT106		G
ECU-27231		Chimborazo Collection B	373	202	202	197	197	223	195	173	161	154	154	184	184	408	402	304	295	G371
			374	196	179	197	197	232	223	173	161	160	145	211	181	421	396	301	295	G372
	Q96		375	179	179	221	197	223	223	161	161	160	145	211	211	402	402	295	295	G373
			376	182	179	221	197	195	192	173	161	160	145	184	181	402	402	295	295	G374
			377	182	182	224	197	217	195	173	161	160	145	211	211	384	384	304	295	G375
			378	182	179	224	197	226	226	173	161	160	145	211	211	421	390	304	295	G376
			379	182	179	224	197	223	195	173	161	160	145	211	211	390	378	304	295	G377
ECU-27232	Q97		380	179	179	197	197	220	220	173	173	160	160	211	181	421	421	301	301	G378
			381	182	182	224	197	220	211	173	173	160	160	184	184	440	440	304	292	G379
			382	176	176	224	197	195	195	173	161	160	160	208	181	440	440	304	292	G380
			383	182	179	197	197	226	226	173	173	160	160	211	211	421	421	301	301	G381
			384	182	182	224	197	223	195	173	173	160	160	211	184	440	384	304	292	G382
ECU-27233	Q98		385	182	179	197	197	223	223	173	173	160	157	211	211	381	381	301	295	G383
			386	173	173	221	197	226	195	188	161	160	148	211	208	402	360	301	295	G384
			387	182	182	221	197	226	195	161	161	160	148	211	184	433	433	301	295	G385
			388	196	182	221	197	226	226	161	161	160	148	211	211	402	381	301	295	G386
			389	182	176	197	197	223	214	173	161	160	157	208	181	421	421	301	295	G387
ECU-27234	Q99		390	176	176	197	197	232	195	188	161	160	160	208	208	402	402	295	295	G388
			391	179	176	197	197	232	195	188	161	160	160	208	208	402	402	295	295	G389
			392	182	179	221	197	223	223	173	173	160	160	211	211	381	381	298	295	G390
			393	179	176	197	197	232	232	161	161	160	160	208	208	402	402	295	295	G391
ECU-27235	Q100		394	176	176	200	197	226	223	173	173	160	151	208	184	402	402	295	295	G392
			395	182	179	218	209	223	220	185	161	160	160	211	211	384	366	298	295	G393
			396	196	182	200	197	226	220	185	161	160	151	211	181	421	378	295	295	G394
			397	182	182	200	197	226	223	173	161	160	151	211	181	402	366	295	295	G395
			398	182	176	200	197	226	226	173	161	160	151	211	211	421	384	295	295	G396
ECU-27236	Q101		399	196	176	200	200	220	195	188	161	160	160	208	181	390	366	298	298	G397
			400	196	196	200	200	195	195	161	161	160	160	181	181	366	366	298	298	G398
ECU-27237	Q102		401	182	176	197	197	195	195	188	161	160	160	184	184	421	366	301	295	G399
			402	182	182	197	197	195	195	161	161	160	148	184	181	378	378	304	301	G400
			403	182	182	197	197	195	195	161	161	160	160	184	184	421	378	301	295	G401
			404	182	182	197	197	195	195	161	161	160	148	208	208	402	366	304	301	G402
			405	182	179	197	197	195	195	185	161	160	148	205	181	421	378	304	301	G403
ECU-27238	Q103		406	173	173	221	221	226	226	182	182	148	148	181	181	372	372	301	292	G404

Supplementary Table S1. Continued.

INIAP GB Code *	Analysis Code	Province Collection	No.	1_QAAT022		2_QAAT024		3_QAAT050		4_QAAT070		5_QAAT076		6_QAAT097		7_QAAT100		8_QAAT106		G
		Chimborazo Collection B	407	182	182	209	209	226	226	161	161	160	160	208	208	372	372	292	292	G405
			408	185	182	221	221	226	226	182	182	148	148	211	184	372	372	301	292	G406
ECU-27239	Q104		409	182	179	209	209	226	226	185	185	160	160	211	181	366	366	301	301	G407
			410	185	182	209	209	226	226	185	185	160	151	208	208	366	366	304	301	G408
			411	185	185	209	209	226	226	185	185	160	151	211	208	366	366	304	301	G409
			412	185	182	209	209	226	226	185	185	160	151	211	208	366	366	304	301	G410
ECU-27240	Q105		413	182	182	227	221	211	211	182	182	160	160	181	181	372	372	298	295	G411
			414	182	182	209	200	226	226	161	161	160	151	181	181	366	366	298	295	G412
			415	182	182	209	200	226	211	188	161	160	151	181	181	366	366	298	295	G413
			416	182	182	227	221	220	211	182	164	160	160	181	181	372	366	298	295	G414
ECU-27241	Q106		417	182	182	200	200	211	211	161	161	160	160	211	211	366	366	301	301	G415
			418	182	182	221	221	226	226	161	161	151	151	208	208	366	366	301	301	G416
			419	182	182	221	221	220	220	161	161	151	151	208	208	366	366	301	301	G417
			420	173	173	200	200	211	211	185	185	160	160	181	181	366	366	301	301	G418
ECU-27243	Q107		421	182	182	197	197	226	220	173	161	160	154	211	181	427	366	295	295	G419
			422	182	182	215	197	226	226	173	173	160	160	211	181	427	402	304	304	G420
			423	188	182	197	197	235	195	188	161	160	154	181	181	366	366	295	295	G421
			424	182	182	197	197	220	220	161	161	160	154	184	184	381	381	295	295	G422
			425	182	182	215	197	232	195	188	188	160	160	181	181	396	390	304	304	G423
ECU-27244	Q108		426	176	176	197	197	232	232	182	182	160	160	211	208	372	372	295	295	G424
			427	182	182	215	197	220	220	161	161	154	145	184	184	381	381	304	295	G425
			428	199	182	215	197	232	220	185	161	154	145	208	181	378	378	304	295	G426
			429	199	182	215	197	226	226	188	188	154	145	184	181	384	378	304	295	G427
			430	182	182	215	197	220	220	161	161	154	145	184	184	384	384	304	295	G428
ECU-27245	Q109		431	196	182	200	197	229	220	188	161	160	151	211	181	421	402	304	301	G429
			432	199	182	200	197	229	220	188	173	160	151	193	181	421	366	304	301	G430
			433	199	182	200	197	229	229	188	188	160	151	193	181	421	366	304	301	G431
			434	199	182	200	197	232	226	173	161	160	151	193	181	366	366	304	301	G432
			435	182	182	221	197	229	217	173	161	160	160	193	181	421	384	295	295	G433
ECU-27247	Q110		436	199	193	209	209	226	195	188	188	160	160	181	181	366	366	301	301	G434
			437	176	176	197	197	217	217	173	173	160	160	193	193	421	421	301	301	G435
			438	202	202	209	209	214	214	188	188	160	160	190	190	366	366	301	301	G436
			439	196	196	209	209	223	223	188	188	160	160	208	208	381	381	301	301	G437
			440	199	193	209	209	195	195	188	188	160	160	181	181	366	366	301	301	G438

Supplementary Table S1. Continued.

INIAP GB Code *	Analysis Code	Province Collection	No.	1_QAAT022		2_QAAT024		3_QAAT050		4_QAAT070		5_QAAT076		6_QAAT097		7_QAAT100		8_QAAT106		G
ECU-27248	Q111	Chimborazo Collection B	441	182	176	197	197	195	195	161	161	160	160	181	181	427	427	295	295	G439
			442	176	176	197	197	217	217	173	173	160	160	193	193	421	421	295	295	G440
			443	182	179	197	197	223	195	173	161	160	160	208	184	396	396	295	295	G441
			444	193	182	197	197	195	195	173	173	160	160	184	184	408	372	295	295	G442
			445	193	193	197	197	226	195	173	161	160	160	187	187	366	366	295	295	G443
ECU-27249	Q112		446	179	179	197	197	223	223	173	161	160	148	211	211	372	372	304	304	G444
			447	196	196	197	197	223	214	161	161	160	148	211	208	372	372	304	304	G445
			448	196	179	197	197	214	214	173	161	160	148	211	208	372	372	304	304	G446
			449	182	182	197	197	195	195	161	161	160	160	208	208	396	396	295	295	G447
			450	196	196	197	197	214	195	173	161	160	148	211	211	384	372	304	304	G448
ECU-27250	Q113		451	179	179	197	197	226	195	161	161	160	154	211	181	396	384	301	301	G449
			452	182	179	197	197	195	195	161	161	154	154	211	187	396	366	301	301	G450
			453	179	179	197	197	195	195	161	161	154	154	181	181	396	396	301	301	G451
			454	196	196	197	197	195	195	161	161	154	154	208	181	396	396	301	301	G452
			455	196	179	197	197	195	195	188	173	160	160	211	181	402	402	301	301	G453
ECU-27252	Q114		456	173	173	224	197	223	223	185	173	160	160	208	208	421	421	295	295	G454
			457	196	196	197	197	217	195	161	161	160	151	181	181	396	378	295	292	G455
			458	193	193	197	197	226	226	161	161	160	151	211	181	378	378	295	292	G456
			459	193	182	197	197	226	220	173	161	160	151	211	181	384	378	295	292	G457
ECU-27253	Q115		460	196	196	215	197	220	195	161	161	151	151	211	211	384	384	295	295	G458
			461	182	182	209	209	195	195	161	161	151	151	211	211	357	357	301	301	G459
			462	196	196	215	197	195	195	161	161	151	151	211	211	384	384	295	295	G460
ECU-27254	Q116		463	182	182	221	221	220	220	161	161	160	160	208	208	384	384	289	289	G461
			464	182	182	221	221	226	223	173	161	160	160	208	208	384	378	301	289	G462
			465	182	182	221	221	226	226	161	161	160	160	208	208	378	378	301	289	G463
			466	182	182	221	221	226	223	173	161	160	160	208	181	378	357	301	289	G464
ECU-27256	Q117		467	182	182	215	215	220	220	188	188	151	151	211	211	396	366	304	304	G465
			468	182	176	224	197	220	195	173	161	160	160	184	181	421	384	304	304	G466
			469	182	182	215	215	220	211	188	188	151	151	181	181	396	378	304	304	G467
			470	182	182	215	215	211	211	188	188	151	151	211	211	357	357	304	304	G468
			471	185	185	224	197	220	220	173	173	160	160	208	184	402	384	304	304	G469
ECU-27257	Q118		472	173	173	221	221	195	195	188	173	160	160	184	181	384	384	301	295	G470
			473	173	173	221	221	226	223	188	161	160	160	184	181	408	384	301	295	G471
			474	173	173	221	221	220	195	161	161	160	160	208	208	396	396	301	295	G472

Supplementary Table S1. Continued.

INIAP GB Code *	Analysis Code	Province Collection	No.	1_QAAT022		2_QAAT024		3_QAAT050		4_QAAT070		5_QAAT076		6_QAAT097		7_QAAT100		8_QAAT106		G
		Chimborazo Collection B	475	173	173	221	221	195	195	188	161	160	160	184	181	366	366	301	295	G473
			476	182	182	221	221	220	195	173	161	160	160	208	208	396	396	301	295	G474
ECU-27258	Q119		477	182	179	227	197	195	195	161	161	163	160	211	184	408	384	295	295	G475
			478	193	193	197	197	226	226	173	173	160	160	181	181	384	384	298	298	G476
			479	193	193	197	197	226	226	173	173	160	160	184	181	384	384	298	298	G477
			480	179	179	227	197	195	195	194	194	163	160	211	190	408	408	295	295	G478
HDEN-67	Q120		481	182	179	215	215	214	211	161	161	160	142	184	184	366	366	301	301	G479
			482	179	179	215	215	217	195	173	161	160	142	184	184	378	378	301	301	G480
			483	173	173	215	197	220	220	161	161	160	154	181	181	384	372	301	301	G481
			484	182	173	215	197	220	211	161	161	160	154	190	184	384	366	301	301	G482
			485	179	179	215	215	220	220	173	161	160	142	181	181	372	372	301	301	G483
ECU-27259	Q121		486	199	182	197	197	195	195	173	161	160	160	208	181	396	384	295	295	G484
			487	199	182	197	197	195	195	188	161	160	160	181	181	402	402	295	295	G485
			488	199	196	197	197	226	195	161	161	160	160	202	181	402	402	295	295	G486
			489	196	196	200	197	226	198	173	161	160	160	211	181	402	366	298	295	G487
			490	196	185	200	197	226	226	161	161	160	160	196	181	402	384	298	295	G488
ECU-27260	Q122		491	196	176	197	197	223	220	173	161	160	160	211	181	421	402	304	295	G489
			492	196	182	197	197	226	220	173	173	160	160	211	181	421	402	304	295	G490
			493	196	182	197	197	226	220	185	161	160	160	211	181	421	372	304	295	G491
			494	179	179	197	197	195	195	161	161	148	148	196	196	421	421	295	295	G492
			495	199	185	197	197	195	195	161	161	148	148	181	181	421	402	295	295	G493
ECU-27261	Q123		496	196	182	221	197	217	217	173	173	160	160	211	181	415	415	301	295	G494
			497	196	182	221	197	226	226	173	161	160	160	181	181	357	357	301	295	G495
			498	196	196	221	197	217	217	173	173	160	160	211	211	415	415	301	301	G496
			499	182	182	197	197	226	226	173	161	151	151	181	181	357	357	295	295	G497
ECU-27262	Q124		500	179	179	197	197	195	195	173	161	160	160	208	208	433	433	298	295	G498
			501	182	179	197	197	226	195	161	161	160	160	208	184	440	433	298	295	G499
			502	196	173	197	197	220	211	173	161	160	160	214	184	402	384	298	295	G500
			503	179	173	197	197	195	195	173	161	160	160	208	208	433	421	298	295	G501
			504	182	179	221	197	232	195	185	161	163	160	211	205	427	372	298	295	G305
ECU-27263	Q125		505	185	173	221	197	226	220	161	161	160	160	181	181	421	372	292	295	G502
			506	182	179	221	197	220	195	161	161	160	160	181	181	408	396	304	295	G503
			507	182	182	221	197	220	195	161	161	160	160	181	181	390	390	304	295	G504
			508	196	179	221	197	220	195	161	161	160	160	208	181	421	421	304	295	G302

Supplementary Table S1. Continued.

INIAP GB Code *	Analysis Code	Province Collection	No.	1_QAAT022		2_QAAT024		3_QAAT050		4_QAAT070		5_QAAT076		6_QAAT097		7_QAAT100		8_QAAT106		G
ECU-27264	Q126	Chimborazo Collection B	509	182	182	197	197	220	220	188	161	160	160	196	196	366	366	295	295	G505
			510	182	176	197	197	195	195	161	161	160	160	196	196	366	366	295	295	G506
			511	182	182	197	197	220	217	188	188	160	154	181	181	402	402	295	295	G507
ECU-27265	Q127		512	185	185	197	197	220	220	173	173	160	160	208	208	427	421	295	295	G508
			513	182	179	197	197	220	220	173	173	160	160	211	208	427	421	295	295	G509
ECU-27266	Q128		514	182	182	200	200	220	220	161	161	160	160	211	211	366	366	301	301	G510
			515	182	182	200	200	211	211	161	161	160	160	181	181	366	366	301	301	G511
			516	182	182	200	200	211	211	182	182	160	160	181	181	366	357	301	301	G512
			517	182	179	200	197	195	195	161	161	160	154	181	181	421	384	301	295	G513
ECU-27267	Q129		518	196	182	200	197	223	211	185	161	160	160	211	181	402	378	304	295	G514
			519	196	196	197	197	195	195	173	161	160	160	181	181	402	402	295	295	G515
			520	182	179	197	197	223	220	161	161	160	160	208	181	402	402	295	295	G516
			521	196	196	197	197	223	223	161	161	160	160	211	211	402	402	295	295	G517
HDEN-79	Q130		522	196	196	215	215	223	223	161	161	160	160	208	208	366	366	301	301	G518
			523	182	182	200	197	220	211	188	161	160	160	190	181	396	357	304	295	G519
			524	176	176	215	215	195	195	188	188	160	160	208	181	421	384	301	301	G520
ECU-27268	Q131		525	199	182	221	209	211	211	188	161	160	160	208	190	366	366	295	295	G521
			526	182	176	221	209	229	220	161	161	160	160	190	181	366	366	295	295	G522
			527	173	173	221	209	220	195	182	182	163	154	181	181	402	366	301	295	G523
			528	185	182	221	209	211	195	161	161	163	154	190	181	384	357	301	295	G524
			529	182	182	221	209	195	195	182	161	160	160	214	181	366	366	295	295	G525
ECU-27269	Q132		530	182	179	197	197	226	226	188	173	160	160	211	208	396	366	304	301	G526
			531	185	182	197	197	226	211	173	173	160	160	211	208	384	366	304	301	G527
			532	182	182	197	197	226	226	161	161	160	160	211	208	384	366	304	301	G528
			533	182	179	197	197	195	195	173	161	163	160	208	181	396	396	301	301	G529
ECU-27270	Q133		534	176	176	197	197	226	195	182	182	166	166	208	208	402	402	301	301	G530
			535	193	193	221	221	223	223	173	173	160	148	181	181	396	396	295	295	G531
			536	193	193	221	221	226	226	173	173	160	148	181	181	396	396	295	295	G532
			537	196	179	221	221	226	226	176	176	160	148	208	208	396	396	295	295	G533
			538	182	182	221	221	211	211	161	161	160	148	208	208	396	396	295	295	G534
ECU-27271	Q134		539	199	196	200	197	232	195	161	161	160	148	181	181	402	366	295	295	G535
			540	182	182	221	221	235	211	161	161	148	148	208	208	396	396	295	295	G536
			541	179	179	200	197	195	195	161	161	160	148	181	181	381	381	295	295	G537
			542	205	205	200	197	223	223	161	161	160	148	211	211	357	357	295	295	G538

Supplementary Table S1. Continued.

INIAP GB Code *	Analysis Code	Province Collection	No.	1_QAAT022		2_QAAT024		3_QAAT050		4_QAAT070		5_QAAT076		6_QAAT097		7_QAAT100		8_QAAT106		G
ECU-27272	Q135	Chimborazo Collection B	543	182	182	197	197	195	195	161	161	160	160	202	202	415	415	295	295	G539
			544	182	173	197	197	223	223	185	173	160	160	205	184	402	384	295	295	G540
			545	193	193	197	197	220	220	161	161	160	160	181	181	421	402	301	301	G541
ECU-27273	Q136		546	193	182	221	197	195	192	161	161	151	151	208	208	402	378	304	295	G542
			547	185	179	197	197	232	232	173	161	160	160	208	181	402	402	298	295	G543
			548	199	182	221	197	232	220	185	161	151	151	211	208	402	378	304	295	G544
			549	196	182	197	197	220	220	161	161	160	160	211	208	357	357	298	295	G545
			550	185	179	197	197	232	211	173	161	160	160	208	208	402	357	298	295	G546
ECU-27275	Q137		551	196	182	197	197	220	198	185	161	160	160	211	181	402	366	301	301	G547
			552	196	182	197	197	220	198	161	161	160	160	211	181	366	357	301	301	G548
			553	179	179	197	197	195	195	161	161	160	160	181	181	381	381	301	301	G549
			554	199	182	197	197	226	198	173	173	160	160	211	181	421	402	301	295	G550
ECU-27276	Q138		555	182	173	221	221	229	207	185	161	160	160	211	211	396	366	332	332	G551
			556	182	173	221	221	207	207	185	161	160	160	211	181	396	366	295	295	G552
			557	182	182	221	221	229	207	185	185	160	160	211	181	396	366	295	295	G553
			558	173	173	221	221	229	207	185	161	160	160	211	181	396	366	295	295	G554
			559	182	173	221	221	207	207	161	161	160	160	211	211	396	366	332	332	G555
ECU-27277	Q139		560	182	182	221	197	223	223	185	185	160	160	181	181	396	390	295	295	G556
			561	182	182	221	197	226	223	173	173	160	160	181	178	396	390	295	295	G557
			562	182	182	221	197	226	226	188	185	160	160	181	181	396	396	295	295	G558
			563	182	176	221	197	226	223	173	173	160	160	181	178	396	390	295	295	G559
ECU-27278	Q140		564	196	196	197	197	223	223	161	161	160	160	211	211	402	402	295	295	G517
			565	196	196	197	197	226	226	161	161	160	160	211	211	384	366	295	295	G560
			566	196	196	197	197	226	226	161	161	160	160	181	181	366	366	295	295	G561
ECU-27279	Q141		567	196	196	197	197	226	211	161	161	160	160	184	178	402	402	301	295	G562
			568	196	176	197	197	211	211	161	161	160	160	208	208	384	384	295	295	G563
			569	196	182	197	197	211	211	161	161	160	160	184	178	402	402	301	295	G564
			570	196	196	197	197	223	211	161	161	160	160	208	208	384	384	295	295	G565
			571	179	179	197	197	195	195	161	161	160	160	181	181	384	384	295	295	G566
ECU-27280	Q142		572	193	193	197	197	195	195	161	161	160	160	181	181	402	402	295	295	G567
			573	193	193	197	197	195	195	161	161	160	160	211	181	384	384	295	295	G568
			574	196	196	221	197	195	195	173	161	160	145	211	208	421	421	295	295	G569
ECU-27281	Q143		575	182	182	197	197	226	223	173	161	160	145	205	181	408	384	301	301	G570
			576	182	182	197	197	223	220	188	161	160	157	208	208	366	366	295	295	G571

Supplementary Table S1. Continued.

INIAP GB Code *	Analysis Code	Province Collection	No.	1_QAAT022		2_QAAT024		3_QAAT050		4_QAAT070		5_QAAT076		6_QAAT097		7_QAAT100		8_QAAT106		G
		Chimborazo Collection B	577	182	176	197	197	220	220	161	161	160	157	208	184	402	402	295	295	G572
			578	182	182	197	197	223	220	185	161	160	157	211	181	366	366	295	295	G573
			579	182	182	197	197	223	220	188	161	160	157	208	181	366	366	295	295	G574
ECU-27282	Q144		580	182	182	218	218	223	223	161	161	160	148	181	181	372	372	301	295	G575
			581	196	185	218	218	220	220	188	173	160	148	181	181	402	396	301	295	G576
			582	196	196	218	218	220	220	173	161	160	148	208	208	405	402	301	295	G577
			583	182	182	218	218	195	195	161	161	160	160	181	181	427	427	304	304	G578
			584	196	173	218	218	229	229	161	161	160	160	208	208	433	433	304	304	G579
ECU-27283	Q145		585	182	182	197	197	223	223	188	188	160	148	181	181	372	372	301	301	G580
			586	176	176	221	221	195	195	161	161	160	160	211	184	384	384	301	301	G581
			587	182	182	221	221	223	223	161	161	160	160	181	181	372	372	301	301	G582
ECU-27284	Q146		588	182	179	197	197	223	220	188	161	160	160	181	181	396	372	301	301	G583
			589	182	179	197	197	223	220	188	161	160	160	208	181	396	372	301	301	G584
			590	196	179	221	197	232	192	173	161	160	160	181	181	384	372	301	301	G585
			591	182	179	221	197	229	195	161	161	160	160	184	184	408	366	301	301	G586
ECU-27285	Q147		592	196	182	197	197	223	195	173	161	160	148	211	181	408	384	301	295	G587
			593	182	182	197	197	226	223	161	161	160	148	202	181	408	402	295	295	G588
			594	182	179	197	197	223	223	161	161	160	148	208	184	402	378	301	295	G589
			595	196	182	197	197	223	223	173	161	160	148	208	181	384	384	301	295	G590
			596	196	176	197	197	229	223	173	173	160	148	211	181	408	384	295	295	G591
ECU-27286	Q148		597	182	182	221	197	207	195	188	161	160	148	184	181	421	396	301	301	G592
			598	182	182	221	197	226	195	161	161	160	148	184	181	421	372	301	301	G593
			599	182	182	197	197	195	195	161	161	160	148	184	184	421	421	301	295	G594
			600	182	182	197	197	220	195	173	161	160	148	184	184	421	421	301	295	G595
			601	182	179	197	197	223	195	188	161	160	148	184	181	421	402	301	295	G596
ECU-27287	Q149		602	173	173	197	197	195	195	161	161	151	151	196	196	408	408	301	301	G597
			603	179	173	218	218	220	220	161	161	160	160	211	196	408	357	301	295	G598
			604	179	173	218	218	220	195	161	161	160	160	211	211	408	357	301	295	G599
			605	179	173	218	218	220	195	161	161	160	160	196	196	408	357	301	295	G600
			606	179	179	218	218	220	220	161	161	160	160	211	211	408	357	301	295	G601
ECU-27288	Q150		607	182	182	224	197	226	226	173	173	166	145	211	181	408	408	295	295	G602
			608	182	182	224	197	195	195	161	161	166	145	181	181	408	408	295	295	G603
			609	179	173	197	197	220	195	188	161	160	160	196	181	421	415	304	304	G604
			610	182	182	224	197	220	220	173	170	166	145	211	181	408	402	295	295	G605

Supplementary Table S1. Continued.

INIAP GB Code *	Analysis Code	Province Collection	No.	1_QAAT022		2_QAAT024		3_QAAT050		4_QAAT070		5_QAAT076		6_QAAT097		7_QAAT100		8_QAAT106		G
ECU-27289	Q151	Chimborazo Collection B	611	179	173	197	197	220	195	188	161	166	160	196	181	421	415	304	304	G606
			612	196	179	209	197	220	220	173	173	160	160	181	181	415	366	295	295	G607
			613	196	179	209	197	220	220	188	188	160	160	181	181	415	415	295	295	G608
ECU-27290	Q152		614	182	173	197	200	211	195	188	161	160	160	193	193	408	408	298	292	G609
			615	173	173	197	200	211	195	188	188	160	160	193	193	366	366	304	295	G610
			616	182	182	197	200	226	195	173	161	160	160	211	181	402	402	298	292	G611
ECU-27291	Q153		617	196	173	209	197	195	195	188	161	160	145	211	211	384	366	301	295	G612
			618	199	193	209	197	220	195	188	161	160	145	208	193	427	357	301	295	G613
			619	196	182	218	197	226	195	188	188	166	160	208	208	402	366	304	295	G614
ECU-27292	Q154		620	182	182	218	197	214	214	188	188	166	160	205	181	384	384	304	295	G615
			621	182	182	209	197	217	195	188	161	160	145	211	193	396	357	301	295	G616
ECU-27293	Q155		622	182	179	224	224	195	195	185	161	160	148	211	196	421	366	295	295	G617
			623	182	182	224	200	220	195	185	185	160	148	205	181	408	384	304	295	G618
			624	182	182	224	200	226	195	185	185	160	148	211	196	402	366	304	295	G619
			625	196	179	200	197	220	211	161	161	166	160	211	211	427	427	298	295	G620
ECU-27294	Q156		626	182	182	197	197	226	226	161	161	160	148	181	181	402	357	301	301	G621
			627	182	182	197	197	226	226	161	161	160	148	181	181	427	427	301	301	G622
			628	182	182	197	197	226	195	161	161	160	148	181	181	427	427	301	301	G623
			629	205	199	218	200	226	198	185	161	160	148	181	181	427	384	301	295	G624
			630	182	182	218	200	223	217	161	161	160	148	190	190	402	402	301	295	G625
ECU-27295	Q157		631	176	176	197	197	223	217	161	161	160	148	208	184	421	408	295	295	G626
			632	185	179	197	197	211	195	173	161	160	148	211	181	402	384	301	301	G627
			633	182	182	197	197	226	195	161	161	160	148	181	181	372	372	301	301	G628
			634	196	196	197	197	226	195	161	161	160	148	181	181	372	372	301	301	G629
			635	185	173	197	197	195	195	161	161	160	148	184	181	408	408	301	301	G630
ECU-27296	Q158		636	196	179	221	197	223	195	161	161	160	160	208	184	421	408	301	298	G631
			637	173	173	221	197	220	220	161	161	160	160	184	184	396	366	301	298	G632
			638	173	173	197	197	220	220	161	161	160	148	208	208	421	421	295	295	G633
			639	182	179	197	197	220	195	161	161	160	148	211	208	402	396	295	295	G634
ECU-27297	Q159		640	196	182	197	197	223	220	173	161	160	148	211	181	421	402	301	295	G635
			641	182	182	197	197	226	223	173	161	160	148	211	181	402	402	301	295	G636
			642	182	179	197	197	223	220	173	161	160	148	211	181	421	402	301	295	G637
			643	185	182	197	197	223	220	161	161	160	148	211	181	421	402	301	295	G638
			644	182	182	197	197	195	195	173	173	160	148	181	181	366	366	295	295	G639

Supplementary Table S1. Continued.

INIAP GB Code *	Analysis Code	Province Collection	No.	1_QAAT022		2_QAAT024		3_QAAT050		4_QAAT070		5_QAAT076		6_QAAT097		7_QAAT100		8_QAAT106		G
ECU-27298	Q160	Chimborazo Collection B	645	182	182	197	197	220	220	161	161	160	157	211	211	421	421	304	295	G640
			646	182	182	197	197	195	195	161	161	160	160	181	181	366	366	304	304	G641
			647	182	182	197	197	232	232	161	161	160	157	181	181	366	366	304	295	G642
			648	182	182	197	197	214	226	173	161	160	160	181	181	421	366	304	304	G643
			649	182	182	197	197	195	195	161	161	160	157	211	181	366	366	304	295	G644
ECU-27299	Q161		650	176	176	221	221	220	220	161	161	160	160	208	208	402	402	301	301	G645
			651	185	176	221	197	220	217	173	161	160	160	208	181	421	402	301	295	G646
ECU-27300	Q162		652	182	182	197	197	220	220	161	161	160	160	211	211	421	421	295	295	G647
			653	182	182	197	197	220	220	173	173	160	160	211	211	421	421	295	295	G648
			654	190	182	197	197	220	220	173	161	160	160	208	208	421	421	295	295	G649
ECU-27301	Q163		655	193	193	197	197	220	220	161	161	163	163	211	211	421	421	295	295	G650
			656	182	182	197	197	226	220	161	161	160	160	211	211	421	421	295	295	G651
			657	190	190	197	197	220	220	161	161	163	163	202	181	402	402	295	295	G652
			658	190	190	197	197	220	220	161	161	160	160	202	202	402	402	295	295	G653
			659	190	182	197	197	226	226	161	161	160	160	211	184	421	421	295	295	G654
ECU-27302	Q164		660	196	173	209	209	195	195	182	161	157	157	184	184	402	366	295	295	G655
			661	179	179	221	197	195	195	161	161	163	160	211	211	378	378	295	295	G656
			662	179	179	221	197	195	192	161	161	163	160	211	211	378	378	295	295	G657
			663	182	182	209	209	217	217	161	161	157	157	181	181	366	366	295	295	G658
			664	196	196	209	209	195	195	182	182	157	157	184	184	402	366	295	295	G659
ECU-27303	Q165		665	179	179	221	221	226	195	173	173	145	145	181	181	421	378	295	295	G660
			666	213	179	221	197	226	195	161	161	154	145	196	196	378	378	304	304	G661
			667	213	179	221	197	226	195	161	161	154	145	196	196	421	378	304	304	G662
			668	213	213	221	221	226	195	173	161	145	145	181	181	421	378	295	295	G663
			669	213	213	221	221	195	195	173	173	145	145	181	181	378	378	295	295	G664
ECU-27305	Q166		670	182	182	209	200	226	211	185	161	160	160	211	190	396	366	301	301	G665
			671	179	179	203	200	220	195	161	161	160	160	211	208	390	357	301	301	G666
			672	179	179	203	200	220	220	161	161	160	160	208	208	366	366	301	301	G667
			673	182	182	209	200	220	220	161	161	160	160	211	190	357	357	301	301	G668
			674	179	179	203	200	220	220	161	161	160	160	208	208	421	421	301	301	G669
ECU-27306	Q167		675	182	182	200	197	226	226	185	185	151	151	208	208	421	421	295	295	G670
			676	182	179	200	197	226	211	161	161	160	151	184	184	421	408	295	295	G671
			677	182	179	200	197	211	211	185	185	160	151	208	208	421	408	295	295	G672
			678	196	196	200	197	220	220	161	161	160	151	211	211	402	402	295	295	G673

Supplementary Table S1. Continued.

INIAP GB Code *	Analysis Code	Province Collection	No.	1_QAAT022		2_QAAT024		3_QAAT050		4_QAAT070		5_QAAT076		6_QAAT097		7_QAAT100		8_QAAT106		G
		Chimborazo Collection B	679	182	182	200	197	226	226	185	185	151	151	184	184	421	408	295	295	G674
ECU-27307	Q168		680	179	179	197	197	195	195	173	161	163	160	211	208	421	421	304	295	G675
			681	196	196	197	197	220	195	161	161	160	160	211	211	402	402	304	295	G676
			682	182	179	218	218	226	220	188	173	145	145	181	181	384	384	295	295	G677
			683	196	179	197	197	220	195	161	161	163	160	211	208	421	402	304	295	G678
			684	196	196	197	197	220	220	161	161	163	160	211	208	421	402	304	295	G679
ECU-27308	Q169		685	179	179	215	215	223	223	188	173	160	160	181	181	421	421	295	295	G680
			686	185	179	218	197	220	195	173	173	160	145	208	184	396	372	295	295	G681
			687	196	182	218	197	217	195	173	161	160	145	211	208	421	366	295	295	G682
ECU-27309	Q170		688	185	185	197	197	223	223	188	161	148	148	208	208	384	384	295	295	G683
			689	196	185	197	197	223	223	188	188	148	148	208	181	384	384	295	295	G684
			690	185	185	197	197	223	223	188	188	148	148	208	208	384	384	295	295	G685
ECU-27310	Q171		691	202	202	221	221	229	229	161	161	160	160	211	211	372	372	295	292	G686
			692	176	176	221	221	229	229	161	161	160	160	181	181	372	372	295	295	G687
			693	176	176	221	221	229	229	161	161	160	160	211	211	372	372	295	295	G688
ECU-27311	Q172		694	176	176	197	197	226	226	161	161	160	160	208	208	372	372	295	295	G689
			695	176	176	221	197	229	226	161	161	160	160	181	181	366	366	298	298	G690
			696	176	176	221	197	229	226	161	161	160	160	211	211	366	366	298	298	G691
			697	176	173	224	224	226	226	161	161	160	160	208	208	366	366	295	295	G692
ECU-27312	Q173		698	176	173	224	224	226	195	161	161	160	160	208	208	396	366	295	295	G693
			699	176	173	197	197	220	195	161	161	148	148	208	208	396	396	295	295	G694
ECU-27313	Q174		700	176	176	197	197	195	195	161	161	160	160	208	181	366	366	295	295	G695
			701	176	176	197	197	195	195	161	161	160	160	208	208	396	366	295	295	G696
			702	176	176	197	197	195	195	161	161	160	160	208	208	381	381	295	295	G697
ECU-27314	Q175		703	176	176	197	197	217	195	173	173	160	160	208	208	384	384	295	295	G698
			704	176	176	221	221	220	220	161	161	160	160	181	181	381	381	301	301	G699
			705	176	176	221	221	223	220	161	161	160	160	211	181	421	363	301	301	G700
			706	176	176	197	197	220	195	173	161	160	160	208	208	384	366	295	295	G701
			707	176	176	197	197	220	195	173	161	160	160	208	181	384	366	295	295	G702
ECU-27315	Q176		708	176	176	200	197	220	220	188	185	160	157	211	181	402	384	301	301	G703
			709	176	176	197	197	220	220	161	161	160	160	211	181	402	384	295	295	G704
ECU-27316	Q177		710	176	176	218	197	220	195	173	161	160	160	181	181	390	381	295	295	G705
			711	176	176	221	197	220	195	173	161	160	160	208	208	390	381	295	295	G706
			712	176	176	218	197	220	195	161	161	160	160	208	205	408	384	295	295	G707

Supplementary Table S1. Continued.

INIAP GB Code *	Analysis Code	Province Collection	No.	1_QAAT022		2_QAAT024		3_QAAT050		4_QAAT070		5_QAAT076		6_QAAT097		7_QAAT100		8_QAAT106		G
		Chimborazo Collection B	713	176	176	221	197	232	229	161	161	160	160	208	208	427	421	295	295	G708
ECU-27317	Q178		714	176	176	197	197	226	220	161	161	160	160	184	184	408	408	298	295	G709
			715	176	176	197	197	226	226	173	161	160	160	181	181	408	408	298	295	G710
			716	176	176	197	197	220	195	161	161	160	160	193	181	408	408	298	295	G711
			717	176	176	209	209	226	226	161	161	160	160	205	205	390	390	295	295	G712
ECU-27319	Q179		718	196	196	200	200	211	211	161	161	160	160	208	208	421	421	304	304	G713
			719	196	196	200	200	217	217	173	173	160	160	208	208	378	378	304	304	G714
			720	196	182	209	200	217	195	173	161	160	160	214	208	378	366	304	295	G715
ECU-27319	Q180		721	196	196	209	197	220	211	173	173	160	160	184	184	396	396	298	295	G716
			722	199	188	200	200	220	220	161	161	160	154	181	181	402	402	295	295	G717
			723	188	188	200	200	220	195	161	161	160	154	181	181	402	402	295	295	G718
			724	188	188	200	200	220	220	173	161	160	154	181	181	402	402	295	295	G719
			725	199	188	200	200	220	220	173	161	160	154	181	181	402	402	295	295	G720
ECU-27320	Q181		726	173	173	197	197	232	232	161	161	160	160	211	181	427	427	295	295	G721
			727	182	182	218	218	223	223	173	173	148	148	208	208	366	366	295	295	G722
			728	173	173	197	197	232	232	161	161	160	160	181	181	427	427	295	295	G723
ECU-27321	Q182		729	199	182	197	197	232	195	188	161	160	157	211	211	366	366	298	295	G724
			730	196	182	197	197	220	195	161	161	160	157	205	181	378	366	298	295	G725
			731	182	182	197	197	220	220	161	161	160	160	184	184	402	378	298	298	G726
			732	182	182	197	197	220	220	161	161	160	160	184	181	378	378	298	298	G727
HDEN-183	Q183		733	199	196	221	215	223	195	188	188	160	160	208	208	402	372	304	295	G728
			734	182	182	215	197	232	232	194	173	160	145	181	181	372	372	295	295	G729
			735	199	199	221	215	232	223	188	161	160	160	181	181	415	402	304	295	G730
			736	176	176	215	197	232	232	194	173	160	145	211	181	402	372	295	295	G731
ECU-27323	Q184		737	173	173	215	197	223	195	173	161	160	160	211	181	408	402	295	295	G732
			738	182	173	215	197	232	195	188	185	160	160	208	175	421	402	295	295	G733
			739	179	179	224	221	232	232	173	173	160	160	211	181	408	408	304	301	G734
			740	179	179	224	221	220	195	182	182	160	160	181	181	402	402	304	301	G735
			741	182	179	224	221	220	220	185	161	160	160	184	181	408	396	304	301	G736
ECU-27324	Q185		742	173	173	221	197	229	220	161	161	163	160	181	181	421	366	295	295	G737
			743	199	199	221	197	223	223	161	161	163	160	181	181	402	402	295	295	G738
			744	182	173	221	197	229	226	185	161	160	160	211	181	402	402	298	295	G739
			745	193	173	221	197	229	229	182	161	160	160	205	181	402	384	298	295	G740
ECU-27325	Q186		746	182	182	197	197	195	195	173	173	160	160	187	187	384	384	304	304	G741

Supplementary Table S1. Continued.

INIAP GB Code *	Analysis Code	Province Collection	No.	1_QAAT022		2_QAAT024		3_QAAT050		4_QAAT070		5_QAAT076		6_QAAT097		7_QAAT100		8_QAAT106		G
		Chimborazo Collection B	747	196	193	197	197	226	226	173	161	160	154	193	181	408	402	295	295	G742
			748	193	182	197	197	223	195	173	173	160	160	211	187	384	384	304	304	G743
			749	199	182	197	197	223	195	173	161	160	160	187	181	402	384	304	304	G744
ECU-27326	Q187		750	179	179	197	197	223	223	173	173	154	148	181	181	384	384	295	295	G745
			751	196	196	197	197	226	226	161	161	160	160	184	184	427	427	295	295	G746
			752	182	182	197	197	226	226	161	161	160	160	211	211	408	408	295	295	G747
			753	185	182	197	197	226	195	188	161	154	148	211	205	384	366	295	295	G748
ECU-27327	Q188		754	196	196	221	221	195	195	188	188	160	160	211	211	421	366	295	295	G749
			755	182	173	221	221	220	220	161	161	151	151	208	208	357	357	301	301	G750
			756	182	182	221	221	211	211	185	185	151	151	208	181	366	366	301	301	G751
			757	182	182	221	221	211	211	161	161	151	151	211	211	366	366	301	301	G752
			758	182	182	221	221	229	229	188	188	160	160	190	190	402	402	295	295	G280
ECU-27363	Q189		759	179	173	224	221	195	195	173	161	160	145	208	181	408	384	295	295	G753
			760	196	182	224	221	226	195	188	161	160	145	208	184	402	384	295	295	G754
			761	196	179	224	224	226	220	161	161	160	160	208	208	421	402	317	317	G755
			762	196	182	224	221	195	195	188	161	160	145	211	181	402	384	295	295	G756
			763	179	179	224	224	232	232	191	191	160	160	208	208	366	366	317	317	G757
ECU-27364	Q190		764	196	196	197	197	226	195	173	173	160	160	184	184	421	402	295	295	G758
			765	199	196	197	197	223	223	161	161	163	163	208	181	396	396	304	304	G759
			766	196	179	197	197	226	226	161	161	160	160	208	181	402	384	295	295	G760
ECU-27365	Q191		767	182	182	221	221	220	220	161	161	160	160	181	181	390	384	295	295	G761
			768	182	182	197	197	223	220	185	161	160	160	211	181	421	396	298	295	G762
			769	182	182	221	221	220	220	161	161	160	160	211	211	421	421	295	295	G763
			770	182	182	197	197	220	220	161	161	160	160	211	211	421	396	298	295	G764
ECU-27367	Q192		771	182	182	197	197	220	220	173	173	160	148	184	184	396	396	301	301	G765
			772	182	182	197	197	220	195	173	173	160	148	184	184	396	396	301	301	G766
			773	185	185	197	197	220	195	173	161	148	148	208	184	396	396	301	301	G767
ECU-27368	Q193		774	193	173	215	197	223	195	161	161	151	145	211	181	402	396	304	295	G768
			775	196	196	215	197	195	195	161	161	151	145	184	184	390	390	304	295	G769
			776	182	179	197	197	226	226	161	161	145	145	208	208	421	408	295	295	G770
			777	182	182	215	197	195	195	161	161	151	145	184	184	366	366	304	295	G771
			778	179	179	197	197	223	223	173	173	145	145	208	208	381	381	295	295	G772
ECU-27369	Q194		779	176	176	197	197	226	211	185	173	160	151	208	181	384	366	301	301	G773
			780	182	176	221	200	220	220	173	173	160	154	211	181	381	381	295	295	G774

Supplementary Table S1. Continued.

INIAP GB Code *	Analysis Code	Province Collection	No.	1_QAAT022		2_QAAT024		3_QAAT050		4_QAAT070		5_QAAT076		6_QAAT097		7_QAAT100		8_QAAT106		G
			781	176	176	197	197	220	220	185	185	160	151	184	184	408	408	301	301	G775
			782	182	182	221	200	220	220	161	161	160	154	208	208	421	421	295	295	G776
			783	182	182	200	200	220	220	185	185	154	154	211	211	366	366	301	301	G777
			784	182	182	200	200	211	211	185	185	154	154	181	181	366	366	301	301	G778
ECU-27370	Q195		785	182	173	200	200	211	211	185	185	154	154	211	208	366	366	301	301	G779
			786	179	179	215	197	226	226	185	185	160	154	181	181	421	421	301	301	G780
			787	182	182	215	197	211	195	185	182	160	154	211	181	408	408	301	301	G781
			788	182	173	197	197	226	226	161	161	154	154	208	181	402	396	295	295	G782
ECU-27371	Q196		789	193	173	197	197	226	195	161	161	154	154	211	181	402	402	295	295	G783
			790	182	182	197	197	220	220	161	161	154	154	208	181	384	384	295	295	G784
			791	196	196	224	218	217	217	161	161	160	154	211	211	384	384	301	301	G785
			792	173	167	221	197	226	220	188	161	160	154	208	208	408	357	298	295	G786
			793	179	179	221	200	226	226	161	161	160	154	184	184	384	384	295	295	G787
ECU-27372	Q197		794	182	167	221	200	226	195	185	161	160	154	211	181	384	366	295	295	G788
			795	179	167	221	200	226	195	185	161	160	154	211	181	384	366	295	295	G789
			796	185	167	221	200	226	226	188	173	160	154	211	181	384	366	295	295	G790
		Chimborazo Collection B	797	193	193	221	197	226	211	161	161	154	142	208	208	402	384	295	295	G791
			798	182	182	221	221	195	195	173	161	160	160	181	181	408	402	295	295	G792
ECU-27373	Q198		799	182	182	221	221	195	195	185	161	160	160	181	181	421	366	295	295	G793
			800	182	182	221	221	195	195	185	161	160	160	181	181	366	366	295	295	G794
			801	176	170	221	221	195	195	161	161	160	160	181	181	366	366	295	295	G795
			802	196	196	197	197	211	211	173	173	160	160	181	181	421	421	295	295	G796
ECU-27374	Q199		803	182	182	197	197	220	220	185	173	160	160	205	205	421	421	295	295	G797
			804	182	182	197	197	220	220	188	188	160	160	211	211	421	421	295	295	G798
			805	182	182	224	224	226	226	173	173	160	160	211	211	421	366	304	295	G799
ECU-27375	Q200		806	182	182	224	224	226	220	173	161	160	160	181	181	402	366	304	295	G800
			807	182	182	221	221	223	220	185	185	160	160	208	184	378	378	304	304	G801
			808	179	176	224	197	223	220	173	161	154	154	184	184	433	390	295	295	G802
			809	173	173	224	197	223	223	173	173	160	160	184	181	390	390	295	295	G803
ECU-27376	Q201		810	173	173	224	197	223	214	173	173	160	160	184	184	396	390	295	295	G804
			811	173	173	224	197	223	214	173	173	160	160	184	181	396	396	295	295	G805
			812	173	173	224	197	220	214	173	161	154	154	184	184	433	390	295	295	G806
ECU-27377	Q202		813	173	170	221	221	226	223	173	161	160	154	181	181	408	396	295	292	G807
			814	176	176	197	197	226	223	173	173	160	154	181	181	421	408	295	292	G808

Supplementary Table S1. Continued.

INIAP GB Code *	Analysis Code	Province Collection	No.	1_QAAT022		2_QAAT024		3_QAAT050		4_QAAT070		5_QAAT076		6_QAAT097		7_QAAT100		8_QAAT106		G
		Chimborazo Collection B	815	173	173	221	221	226	195	173	161	160	154	181	181	408	396	295	292	G809
			816	173	173	197	197	226	223	173	173	160	154	181	181	421	408	295	292	G810
ECU-27378	Q203		817	196	179	197	197	226	226	161	161	160	160	208	208	381	381	295	295	G811
			818	193	182	224	197	226	195	173	161	160	154	208	181	384	357	304	295	G812
			819	199	182	224	197	195	195	173	173	160	154	211	181	384	384	304	295	G813
			820	179	179	197	197	223	220	161	161	160	160	181	181	384	384	295	295	G814
			821	193	193	224	197	226	195	173	173	160	154	208	181	402	384	304	295	G815
ECU-27379	Q204		822	193	193	197	197	195	195	161	161	160	160	211	211	384	384	295	295	G816
			823	193	193	197	197	223	223	173	173	160	160	211	211	402	402	295	295	G817
			824	199	193	197	197	226	223	185	161	160	160	208	181	384	366	295	295	G818
			825	199	199	197	197	223	223	161	161	160	160	208	208	402	402	295	295	G819
			826	173	173	197	197	226	226	173	173	160	160	181	181	390	390	295	295	G820
HDEN-179	Q205		827	173	173	224	224	217	217	185	161	166	166	181	181	378	378	301	301	G821
			828	182	182	197	197	220	220	161	161	160	160	211	211	396	396	295	295	G822
			829	199	182	197	197	220	220	188	161	160	160	211	208	384	384	295	295	G823
			830	176	176	197	197	226	226	188	188	160	160	208	208	366	366	295	295	G824
			831	182	182	224	224	226	226	161	161	166	166	181	181	357	357	301	301	G825
ECU-27334	Q206	Cotopaxi Collection B	832	196	196	224	200	220	195	188	185	154	154	211	181	366	366	304	301	G826
			833	182	182	224	200	195	192	188	185	154	154	211	181	366	366	304	301	G827
			834	182	182	224	224	211	211	188	188	154	154	190	190	378	366	304	304	G828
			835	196	182	224	224	211	195	185	161	154	154	190	181	396	366	304	304	G829
ECU-27335	Q207		836	167	167	200	200	198	198	182	182	154	154	187	187	415	415	329	329	G830
			837	182	182	197	197	223	223	185	161	154	154	208	208	366	366	295	295	G831
			838	182	182	197	197	223	223	182	182	154	154	208	208	402	402	295	295	G832
HDEN-184	Q208		839	185	185	197	197	223	223	185	185	154	154	205	205	396	396	295	295	G833
ECU-27336	Q209		840	202	202	215	215	214	214	188	188	148	148	190	190	396	396	295	295	G834
			841	202	202	215	215	214	214	188	188	148	148	208	208	396	396	295	295	G835
			842	182	182	218	218	226	220	185	185	148	148	208	208	366	366	301	292	G836
ECU-27337	Q210		843	182	179	200	200	220	220	161	161	160	160	211	211	366	357	298	295	G837
			844	182	182	200	200	220	220	161	161	160	154	211	211	366	366	301	301	G838
ECU-27338	Q211		845	210	210	215	197	211	211	173	173	154	148	211	208	415	415	298	295	G839
			846	210	210	215	197	211	207	188	173	154	148	211	211	366	360	298	295	G840
			847	185	185	215	197	207	207	188	173	154	148	211	211	408	366	298	295	G841
			848	182	182	200	200	220	220	161	161	160	160	211	211	366	357	298	295	G842

Supplementary Table S1. Continued.

INIAP GB Code *	Analysis Code	Province Collection	No.	1_QAAT022		2_QAAT024		3_QAAT050		4_QAAT070		5_QAAT076		6_QAAT097		7_QAAT100		8_QAAT106		G
		Cotopaxi Collection B	849	185	182	215	197	211	207	188	173	154	148	211	187	408	366	298	295	G843
ECU-27339	Q212		850	196	182	224	221	220	195	188	173	160	160	208	181	408	402	295	292	G844
			851	193	179	224	221	223	223	161	161	160	160	184	181	421	402	295	292	G845
			852	185	173	224	221	223	223	188	161	160	160	184	181	384	384	295	292	G846
			853	193	182	197	197	214	195	173	161	160	157	211	208	402	384	295	295	G847
			854	193	185	197	197	223	195	161	161	160	157	211	181	402	402	295	295	G848
ECU-27340	Q213		855	196	182	215	197	229	223	161	161	160	160	181	181	402	402	301	301	G849
			856	182	182	215	197	195	195	161	161	160	160	184	184	427	427	301	301	G850
			857	196	196	197	197	223	223	173	161	160	160	208	208	427	421	301	301	G851
			858	196	196	197	197	232	223	173	161	160	160	211	211	421	421	301	301	G852
ECU-27341	Q214		859	176	176	197	197	226	226	161	161	157	157	181	181	415	415	295	295	G853
			860	182	182	197	197	226	226	161	161	157	157	181	181	415	415	295	295	G854
			861	182	182	197	197	226	226	161	161	157	157	181	181	381	381	295	295	G855
ECU-27342	Q215		862	182	182	200	200	195	195	185	185	151	151	211	211	366	366	304	304	G856
			863	182	182	200	200	232	195	188	185	151	151	211	211	357	357	304	304	G857
			864	182	182	215	200	195	195	185	161	160	151	211	181	396	366	304	301	G858
ECU-27343	Q216		865	179	173	221	200	195	195	161	161	151	145	196	181	415	408	304	304	G859
			866	176	176	218	218	226	226	188	161	148	148	208	181	366	366	298	298	G860
			867	176	176	218	218	226	226	188	188	148	148	208	208	366	366	298	298	G861
ECU-27344	Q217		868	199	199	215	215	226	226	173	173	157	157	175	175	402	402	317	317	G862
			869	199	199	224	224	226	226	173	173	160	160	211	208	402	402	317	317	G863
ECU-27345	Q218		870	196	176	197	197	211	211	188	173	160	160	211	205	372	372	295	295	G864
			871	196	196	197	197	211	211	188	173	160	160	211	205	372	372	295	295	G865
			872	196	196	197	197	211	211	188	188	160	160	211	205	372	372	295	295	G866
			873	196	196	197	197	211	211	188	188	160	160	211	211	372	372	295	295	G867
ECU-27346	Q219		874	182	182	218	218	223	223	191	191	160	148	205	181	372	366	295	295	G868
			875	182	179	218	218	223	223	191	191	160	148	205	205	372	372	295	295	G869
			876	179	179	218	218	223	223	191	191	160	148	205	205	372	366	295	295	G870
			877	176	176	218	218	223	211	191	188	160	148	205	181	366	366	295	295	G871
			878	199	199	218	218	207	207	191	188	160	148	205	205	372	372	295	295	G872
ECU-27347	Q220		879	213	176	197	197	220	220	161	161	160	157	211	211	402	402	298	295	G873
			880	182	182	197	197	220	220	185	161	160	157	181	181	408	357	298	295	G874
			881	196	173	197	197	220	195	185	161	163	160	211	184	402	366	298	295	G875
ECU-27348	Q221		882	182	182	200	200	211	211	185	185	160	160	181	181	366	366	301	301	G876

Supplementary Table S1. Continued.

INIAP GB Code *	Analysis Code	Province Collection	No.	1_QAAT022		2_QAAT024		3_QAAT050		4_QAAT070		5_QAAT076		6_QAAT097		7_QAAT100		8_QAAT106		G
		Cotopaxi Collection B	883	182	182	200	200	211	211	185	185	160	160	211	181	390	390	301	301	G877
ECU-27349	Q222		884	182	182	200	200	220	220	161	161	160	160	181	181	366	357	301	301	G878
			885	182	182	200	200	220	220	161	161	160	160	190	181	357	357	301	301	G879
			886	182	182	200	200	220	220	161	161	160	160	181	181	357	357	301	301	G880
			887	193	182	200	200	223	223	161	161	160	160	184	181	366	366	301	301	G881
ECU-27350	Q223		888	182	182	200	200	220	220	161	161	160	160	211	211	408	408	301	301	G882
			889	182	182	200	200	211	211	161	161	160	160	181	181	366	366	301	301	G511
			890	173	173	200	200	220	220	185	185	160	160	181	181	357	357	301	301	G883
			891	182	173	200	200	220	220	185	161	160	160	211	211	366	366	301	301	G884
ECU-27351	Q224		892	196	196	221	221	214	214	188	188	151	148	211	205	366	366	317	317	G885
			893	199	196	221	221	214	214	188	188	151	148	175	175	366	366	317	317	G886
			894	199	199	209	209	214	214	188	188	148	148	211	211	390	390	317	317	G887
ECU-27352	Q225		895	196	196	209	209	214	214	188	188	148	148	205	205	390	390	317	317	G888
			896	199	199	221	221	214	214	188	188	151	151	172	172	372	372	295	295	G889
			897	199	199	221	221	214	214	188	188	151	151	172	172	366	366	295	295	G890
			898	199	199	221	221	214	214	188	188	151	151	172	172	372	366	295	295	G891
			899	199	199	221	221	214	214	188	161	151	151	172	172	366	366	295	295	G892
ECU-27353	Q226		900	202	202	221	221	214	214	188	161	148	148	190	190	366	366	317	317	G893
			901	202	202	221	221	226	226	188	188	148	148	175	175	366	366	317	317	G894
			902	196	196	200	200	223	223	188	188	163	163	208	208	381	381	292	292	G895
			903	202	202	221	221	214	214	188	188	148	148	190	190	366	366	317	317	G896
ECU-27354	Q227		904	196	196	221	197	226	226	188	161	160	148	211	211	384	384	320	320	G897
			905	196	179	221	197	226	226	188	188	160	148	211	181	384	384	320	320	G898
			906	199	182	215	197	232	207	188	188	148	142	211	175	396	384	317	317	G899
ECU-27355	Q228		907	182	182	221	221	207	207	188	188	157	157	217	211	396	396	317	317	G900
			908	199	199	197	197	207	207	188	188	148	148	211	211	396	396	317	317	G901
			909	199	182	197	197	207	207	188	188	148	148	217	211	366	366	317	317	G902
			910	199	199	197	197	207	207	188	188	148	148	211	211	366	366	317	317	G903
ECU-27356	Q229		911	199	199	224	221	226	226	188	188	148	148	175	175	396	396	317	313	G904
			912	199	185	224	221	226	195	188	188	148	148	214	175	366	366	317	313	G905
			913	196	196	224	221	211	211	188	188	148	148	187	187	366	366	317	313	G906
			914	185	185	224	221	226	195	188	188	148	148	214	214	396	366	317	313	G907
ECU-27357	Q230		915	202	185	221	221	229	207	188	188	148	145	211	175	396	372	317	295	G908
			916	185	185	221	221	207	207	188	188	148	145	175	175	372	372	317	295	G909

Supplementary Table S1. Continued.

INIAP GB Code *	Analysis Code	Province Collection	No.	1_QAAT022		2_QAAT024		3_QAAT050		4_QAAT070		5_QAAT076		6_QAAT097		7_QAAT100		8_QAAT106		G
		Cotopaxi Collection B	917	196	196	221	221	211	211	188	188	160	160	187	187	366	366	317	317	G910
			918	185	185	221	221	229	207	188	188	148	145	175	175	372	372	317	295	G911
			919	185	182	221	221	207	207	188	185	160	160	187	181	372	366	317	317	G912
ECU-27358	Q231		920	199	199	221	197	195	195	188	185	148	148	205	181	366	366	317	317	G913
			921	199	196	221	197	207	207	188	188	148	148	205	181	366	366	317	317	G914
			922	199	199	221	221	195	195	188	185	148	148	196	181	372	366	317	313	G915
			923	199	199	221	197	207	207	188	185	148	148	205	181	372	372	317	317	G916
ECU-27359	Q232		924	199	199	197	197	207	207	188	188	160	160	175	175	390	390	295	295	G917
			925	205	199	197	197	207	207	188	188	160	160	175	175	390	390	295	295	G918
ECU-27328	Q233		926	182	182	200	200	220	220	161	161	163	160	181	181	396	396	301	301	G919
			927	182	182	200	200	220	220	161	161	160	160	208	181	366	366	301	301	G920
			928	173	173	200	200	220	220	161	161	160	160	181	181	366	366	301	301	G921
			929	182	182	200	200	220	195	185	161	163	160	181	181	396	357	301	301	G922
			930	182	182	200	200	195	195	185	185	163	160	190	181	366	366	301	301	G923
ECU-27329	Q234	Imbabura Collection B	931	182	182	200	200	211	211	161	161	160	160	208	208	357	357	301	301	G924
			932	182	182	200	200	220	220	161	161	160	160	181	181	357	357	301	301	G880
			933	182	182	200	200	211	211	185	185	160	160	211	211	372	372	301	301	G925
			934	182	182	200	200	211	211	161	161	160	160	211	211	366	366	301	301	G415
ECU-27330	Q235		935	182	182	200	200	211	211	185	185	160	160	211	211	408	408	295	295	G926
			936	173	173	200	200	211	211	185	185	160	160	181	181	372	372	295	295	G927
			937	182	182	200	200	211	211	185	185	160	160	211	211	366	366	295	295	G928
			938	182	182	200	200	220	220	188	188	151	151	181	181	357	357	301	301	G929
ECU-27331	Q236		939	182	182	221	221	195	195	188	185	157	157	208	181	396	396	301	301	G930
			940	182	182	221	221	229	229	161	161	160	160	181	181	366	366	301	301	G931
			941	182	182	221	221	223	223	188	188	157	157	181	181	408	408	301	301	G932
			942	182	182	221	221	220	220	161	161	160	160	211	211	402	366	301	301	G933
			943	182	182	221	221	232	232	185	161	157	157	181	181	396	396	301	301	G934
ECU-27332	Q237		944	199	199	221	221	211	211	194	194	160	160	211	211	396	396	317	317	G935
			945	199	199	221	221	211	198	194	188	160	160	211	211	396	396	317	317	G936
			946	199	199	221	221	198	198	194	188	160	160	211	211	396	396	317	317	G937
ECU-27333	Q238		947	182	182	221	221	198	198	188	161	151	151	181	181	372	357	317	317	G938
			948	182	182	221	221	198	198	188	188	151	151	208	208	372	372	317	317	G939
			949	182	182	221	221	198	198	188	188	160	160	181	181	396	396	301	301	G940
			950	182	182	221	221	198	198	188	188	160	160	181	181	396	372	301	301	G941

Supplementary Table S1. Continued.

INIAP GB Code *	Analysis Code	Province Collection	No.	1_QAAT022		2_QAAT024		3_QAAT050		4_QAAT070		5_QAAT076		6_QAAT097		7_QAAT100		8_QAAT106		G
ECU-27360	Q239	Imbabura Collection B	951	199	193	221	218	211	211	191	188	151	148	211	205	366	366	320	317	G942
			952	199	199	221	218	211	211	188	185	151	148	205	205	366	366	320	317	G943
			953	193	193	218	218	211	211	188	188	151	148	211	211	366	366	317	317	G944
HDEB-217	Q240		954	182	182	209	209	220	211	185	185	163	160	211	211	372	366	301	301	G945
			955	182	182	209	209	220	220	185	185	163	160	211	211	357	357	301	301	G946
			956	196	182	215	200	195	195	185	161	160	160	193	181	366	366	329	301	G947
ECU-27362	Q241		957	179	179	200	200	195	195	185	185	160	160	181	181	366	366	301	301	G948
			958	182	182	200	200	211	211	185	185	160	160	211	181	366	366	301	301	G949
			959	182	182	200	200	195	195	185	185	160	160	211	211	366	366	301	301	G950
ECU-27380	Q242		960	182	182	218	218	220	220	185	185	148	148	208	208	366	366	292	292	G951
ECU-27381	Q243		961	182	182	221	221	220	220	185	185	148	148	181	181	366	366	317	317	G952
			962	199	199	215	215	195	195	191	191	154	154	208	208	366	366	295	295	G953
			963	182	182	221	221	217	211	161	161	148	148	193	190	366	366	317	317	G954
			964	196	196	221	221	211	211	188	188	148	148	181	181	366	366	317	317	G955
			965	182	182	215	215	195	195	185	185	154	154	181	181	366	366	295	295	G956
ECU-27382	Q244		966	182	182	218	200	226	220	194	191	160	151	211	193	366	366	298	295	G957
			967	199	199	215	215	195	195	191	191	154	154	208	208	366	366	295	295	G953
			968	182	182	218	200	226	195	194	194	160	151	211	190	366	366	298	295	G958
			969	182	182	218	200	195	195	194	191	160	151	181	181	366	366	298	295	G959
			970	185	185	215	215	195	195	194	194	154	154	181	181	366	366	295	295	G960
ECU-27383	Q245		971	182	182	200	200	207	207	188	188	163	163	211	211	396	396	295	295	G961
			972	182	182	200	200	226	207	188	161	163	163	181	181	396	366	295	295	G962
			973	182	182	221	197	207	207	188	188	169	160	211	211	402	396	295	295	G963
			974	182	179	221	197	226	195	161	161	169	160	211	181	402	396	295	295	G964
			975	179	179	200	200	207	207	173	161	163	163	211	211	421	421	295	295	G965
ECU-27384	Q246		976	182	182	197	197	226	226	185	185	148	148	181	181	354	354	301	301	G966
			977	182	182	221	221	220	220	185	185	151	151	208	208	366	366	317	317	G967
			978	185	185	197	197	220	220	188	188	148	148	181	181	396	396	301	301	G968
			979	182	182	197	197	226	226	188	188	148	148	181	181	366	366	301	301	G969
			980	182	182	221	221	195	195	185	185	151	151	208	208	366	366	317	317	G970
ECU-27386	Q247		981	185	182	221	197	220	195	188	185	160	160	208	193	366	366	298	295	G971
			982	182	179	197	197	226	226	188	185	148	148	208	181	402	366	301	295	G972
			983	182	182	221	197	195	195	185	185	160	160	184	184	418	366	298	295	G973
			984	182	182	221	197	195	195	185	185	160	160	211	211	366	366	298	295	G974

Supplementary Table S1. Continued.

INIAP GB Code *	Analysis Code	Province Collection	No.	1_QAAT022		2_QAAT024		3_QAAT050		4_QAAT070		5_QAAT076		6_QAAT097		7_QAAT100		8_QAAT106		G
		Imbabura Collection B	985	179	179	197	197	220	195	188	188	148	148	208	181	402	396	301	295	G975
ECU-27387	Q248		986	182	182	197	197	226	226	185	185	151	151	214	214	396	396	295	295	G976
			987	185	185	215	215	226	226	194	194	151	151	211	211	396	396	295	295	G977
			988	182	182	197	197	226	226	188	188	151	151	211	211	396	396	295	295	G978
			989	185	185	215	215	214	214	194	194	151	151	211	211	396	396	295	295	G979
ECU--27389	Q249		990	182	182	221	221	195	195	188	188	160	160	187	187	378	378	295	295	G980
			991	185	185	221	221	195	195	188	188	160	160	187	187	408	378	295	295	G981
			992	182	182	221	221	214	214	188	188	160	160	181	181	372	372	295	295	G982
			993	199	173	200	200	223	211	188	185	160	160	208	181	396	396	317	301	G983
ECU-27390	Q250		994	202	182	221	221	220	211	188	188	160	160	208	208	357	357	313	295	G984
			995	182	182	221	221	220	220	188	188	160	160	184	184	357	357	295	295	G985
ECU-27391	Q251		996	182	182	197	197	195	195	194	194	151	151	181	181	372	372	295	295	G986
			997	185	185	197	197	195	195	194	194	151	151	181	181	366	366	295	295	G987
			998	185	185	215	215	195	195	188	188	151	151	181	181	366	366	295	295	G988
			999	182	182	197	197	195	195	194	194	151	151	208	208	372	372	295	295	G989
ECU-27392	Q252		1000	182	182	221	221	198	195	191	185	148	148	181	181	396	396	301	301	G990
			1001	199	199	215	215	211	211	188	188	160	160	208	208	366	366	295	295	G991
ECU-27393	Q253		1002	188	188	215	215	198	198	188	188	160	160	208	208	366	366	313	313	G992
			1003	202	202	215	215	211	211	188	188	160	160	208	208	366	366	313	313	G993
			1004	182	182	215	215	195	195	185	185	160	160	208	208	366	366	313	313	G994
			1005	185	182	221	221	220	220	188	188	148	148	208	181	372	372	301	301	G995
ECU-27394	Q254		1006	182	182	197	197	220	220	185	185	148	148	190	190	366	366	295	295	G996
			1007	182	182	197	197	226	211	161	161	148	148	208	190	366	357	295	295	G997
			1008	182	182	197	197	220	220	161	161	148	148	181	181	366	357	295	295	G998
			1009	182	182	221	221	220	220	188	188	160	160	199	199	354	354	301	301	G999
			1010	207	207	197	197	195	195	194	194	148	148	187	187	366	366	295	295	G1000
ECU-27395	Q255		1011	182	182	221	197	195	195	185	185	163	148	184	184	396	396	298	295	G1001
			1012	182	182	221	197	226	195	191	185	163	148	208	181	402	354	298	295	G1002
			1013	182	182	221	221	220	220	191	191	166	166	208	208	372	372	295	295	G1003
			1014	179	179	221	221	220	220	191	191	166	166	208	208	372	372	295	295	G1004
			1015	182	182	221	197	220	220	161	161	163	148	211	211	366	366	298	295	G1005
ECU-27396	Q256		1016	182	182	197	197	220	220	191	191	160	160	208	208	372	372	295	295	G1006
			1017	182	182	197	197	195	195	185	185	160	160	184	184	396	396	295	295	G1007
			1018	182	182	200	200	211	211	185	185	160	160	208	190	366	366	301	301	G1008

Supplementary Table S1. Continued.

INIAP GB Code *	Analysis Code	Province Collection	No.	1_QAAT022		2_QAAT024		3_QAAT050		4_QAAT070		5_QAAT076		6_QAAT097		7_QAAT100		8_QAAT106		G
ECU-27397		Imbabura Collection B	1019	182	173	200	200	220	211	185	161	160	160	181	181	366	357	301	301	G1009
	Q257		1020	196	196	200	200	214	214	161	161	151	151	181	181	372	372	301	301	G1010
			1021	182	182	197	197	198	195	185	185	160	157	211	181	372	366	317	301	G1011
			1022	185	182	197	197	211	195	185	185	160	157	181	181	396	366	317	301	G1012
			1023	182	182	197	197	229	195	188	185	160	157	208	181	366	366	317	301	G1013
ECU-27398	Q258		1024	182	182	200	200	220	220	161	161	160	160	181	181	366	357	301	301	G878
			1025	182	182	200	200	211	211	161	161	160	160	181	181	357	357	301	301	G1014
			1026	185	182	200	200	211	211	188	188	160	160	190	181	396	357	301	301	G1015
			1027	182	182	200	200	220	211	185	161	160	160	181	181	357	357	301	301	G1016
			1028	182	182	197	197	220	220	191	191	160	160	208	208	372	372	301	301	G1017
ECU-27399	Q259		1029	182	182	215	197	226	226	185	185	148	148	181	181	396	396	317	304	G1018
			1030	182	182	197	197	226	195	185	185	151	151	214	208	396	390	295	295	G1019
ECU-27400	Q260		1031	185	185	221	197	214	211	191	185	148	148	181	181	396	396	295	295	G1020
			1032	185	185	221	197	214	211	191	185	148	148	193	193	396	366	295	295	G1021
			1033	182	182	221	197	214	211	185	185	148	148	181	181	396	366	295	295	G1022
			1034	193	182	221	221	198	198	185	185	160	157	184	181	396	396	295	295	G1023
			1035	182	182	221	197	211	211	191	185	148	148	193	181	366	366	295	295	G1024
ECU-27401	Q261		1036	182	182	197	197	211	211	161	161	160	160	181	181	366	366	295	295	G1025
			1037	196	173	215	200	220	195	161	161	160	160	211	208	366	366	304	301	G1026
			1038	182	182	215	200	211	195	161	161	160	160	181	181	366	366	304	301	G1027
			1039	182	182	215	200	211	211	161	161	160	160	181	181	366	366	304	301	G1028
ECU-27402	Q262		1040	185	185	221	221	226	226	182	182	151	148	211	211	372	372	295	295	G1029
			1041	182	182	218	218	226	226	185	185	148	148	211	211	372	372	292	292	G1030
			1042	182	182	218	218	220	220	185	185	148	148	208	208	366	366	292	292	G951
			1043	199	185	221	221	226	195	188	182	151	148	211	181	396	372	295	295	G1031
ECU-27404	Q263		1044	182	182	197	197	232	220	188	161	160	151	211	181	396	366	295	295	G1032
			1045	182	182	197	197	195	195	161	161	160	151	181	181	366	366	295	295	G1033
			1046	182	182	197	197	217	217	185	185	148	148	211	211	372	372	295	295	G1034
ECU-27405	Q264		1047	182	182	221	221	226	226	185	185	151	151	211	211	372	372	301	301	G1035
			1048	185	185	221	221	226	226	182	182	151	151	211	211	372	372	301	301	G1036
			1049	182	182	221	221	226	226	185	185	148	148	208	208	366	366	301	301	G1037
ECU-27406	Q265		1050	182	182	209	209	211	195	188	185	163	151	181	181	396	396	304	301	G1038
			1051	182	182	209	209	195	195	188	185	163	151	181	181	396	396	304	301	G1039
			1052	182	182	209	209	220	195	188	161	163	151	190	190	396	366	304	301	G1040

Supplementary Table S1. Continued.

INIAP GB Code *	Analysis Code	Province Collection	No.	1_QAAT022		2_QAAT024		3_QAAT050		4_QAAT070		5_QAAT076		6_QAAT097		7_QAAT100		8_QAAT106		G
ECU-27407		Imbabura Collection B	1053	182	182	209	209	220	211	161	161	160	151	211	193	408	366	301	301	G1041
ECU-27407	Q266	Imbabura Collection B	1054	182	182	197	197	226	226	188	188	160	160	181	181	366	366	301	301	G1042
ECU-27407	Q266	Imbabura Collection B	1055	182	182	197	197	226	226	182	161	160	160	208	208	366	366	301	301	G1043
ECU-27407	Q266	Imbabura Collection B	1056	182	182	197	197	198	198	185	185	160	160	193	193	366	366	301	301	G1044
ECU-27407	Q266	Imbabura Collection B	1057	182	182	197	197	226	226	182	182	160	160	208	208	366	366	301	301	G1045
ECU-27407	Q266	Imbabura Collection B	1058	173	173	209	209	226	223	173	161	160	148	181	181	421	408	295	295	G1046
ECU-27492	Q267	Imbabura Collection B	1059	182	182	200	200	211	211	161	161	151	151	190	190	366	366	301	301	G1047
ECU-27492	Q267	Imbabura Collection B	1060	179	179	215	215	220	220	188	161	160	154	211	211	366	366	301	301	G1048
ECU-27492	Q267	Imbabura Collection B	1061	179	179	215	215	220	220	185	185	160	154	193	193	366	366	301	301	G1049
ECU-27492	Q267	Imbabura Collection B	1062	196	185	200	200	211	211	185	185	151	151	181	181	366	366	301	301	G1050
ECU-27492	Q267	Imbabura Collection B	1063	182	182	200	200	211	211	161	161	151	151	211	211	366	366	301	301	G1051
ECU-27493	Q267	Imbabura Collection B	1064	196	196	200	200	211	211	188	188	154	154	205	205	378	378	317	317	G1052
ECU-27493	Q267	Imbabura Collection B	1065	196	185	221	200	211	195	188	188	154	154	205	205	378	378	317	317	G1053
ECU-27493	Q267	Imbabura Collection B	1066	196	196	221	200	211	195	188	188	154	154	205	205	378	378	317	317	G1054
ECU-27493	Q268	Imbabura Collection B	1067	185	185	197	197	226	226	185	185	160	160	208	208	372	372	295	295	G1055
ECU-0621	INIAP-Tunkahuan			182	182	209	200	220	195	188	185	160	160	193	181	366	366	301	301	G_T1
ECU-0621	INIAP-Tunkahuan			182	182	215	209	220	195	161	161	160	151	211	181	366	366	304	301	G_T2
ECU-0621	INIAP-Tunkahuan			182	182	215	215	220	220	188	188	160	160	193	193	396	396	301	301	G_T3
ECU-0621	INIAP-Tunkahuan			182	182	*215*	*209*	220	220	161	161	*160*	*151*	211	193	366	366	*304*	*301*	G_T4

* A collection code is indicated in italics for accessions in which the farmer did not donate the seeds collected during 2014-2015 to be conserved in the INIAP GB.

Supplementary Table S2. Allele sizes (A; in base pairs) and their frequencies (F) found for each of the eight microsatellite loci analyzed in 1067 quinoa samples. Alleles with frequencies > 0.3 are shown in bold.

No.	1_QAAT022		2_QAAT024		3_QAAT050		4_QAAT070		5_QAAT076		6_QAAT097		7_QAAT100		8_QAAT106	
	**A**	**F**	**A**	**F**	**A**	**F**	**A**	**F**	**A**	**F**	**A**	**F**	**A**	**F**	**A**	**F**
1	167	0.003	194	0.003	192	0.005	**161**	**0.424**	142	0.004	172	0.004	354	0.009	289	0.002
2	170	0.001	**197**	**0.448**	195	0.258	164	0.001	145	0.043	175	0.011	357	0.037	292	0.041
3	173	0.060	200	0.107	198	0.034	167	0.001	148	0.193	178	0.003	360	0.001	**295**	**0.484**
4	176	0.077	203	0.001	201	0.003	170	0.002	151	0.067	**181**	**0.319**	363	0.001	298	0.057
5	179	0.134	209	0.045	204	0.007	173	0.136	154	0.066	184	0.071	366	0.267	301	0.245
6	**182**	**0.404**	212	0.002	207	0.036	176	0.001	157	0.052	187	0.020	372	0.079	304	0.073
7	185	0.055	215	0.072	211	0.094	182	0.038	**160**	**0.533**	190	0.027	378	0.037	307	0.001
8	188	0.015	218	0.042	214	0.030	185	0.134	163	0.028	193	0.020	381	0.020	313	0.010
9	190	0.005	221	0.225	217	0.023	188	0.217	166	0.008	196	0.018	384	0.085	317	0.075
10	193	0.034	224	0.041	220	0.158	191	0.027	169	0.004	199	0.002	387	0.001	320	0.006
11	196	0.109	227	0.005	223	0.106	194	0.017	172	0.003	202	0.008	390	0.030	329	0.004
12	199	0.064	236	0.001	226	0.183	197	0.001			205	0.033	396	0.127	332	0.002
13	202	0.021	239	0.004	229	0.029					208	0.263	402	0.111		
14	205	0.009	245	0.002	232	0.031					211	0.190	405	0.001		
15	207	0.001			235	0.001					214	0.008	408	0.054		
16	210	0.002			238	0.001					217	0.002	411	0.001		
17	213	0.003											415	0.018		
18	219	0.002											418	0.000		
19	235	0.001											421	0.087		
20													427	0.020		
21													433	0.007		
22													440	0.004		
23													446	0.001		
24													458	0.003		

Supplementary Table S3. Genotypes (g) and their frequencies (F) found for each of the eight microsatellite loci analyzed in 1067 quinoa samples. Genotypes with frequencies > 0.2 are shown in bold.

No.	1_QAAT022		2_QAAT024		3_QAAT050		4_QAAT070		5_QAAT076		6_QAAT097		7_QAAT100		8_QAAT106	
	**g**	**F**	**g**	**F**	**g**	**F**	**g**	**F**	**g**	**F**	**g**	**F**	**g**	**F**	**g**	**F**
1	167-167	0.001	194-194	0.002	192-192	0.003	**161-161**	**0.321**	142-142	0.002	172-172	0.004	354-354	0.006	289-289	0.001
2	167-173	0.001	194-224	0.003	192-195	0.004	161-173	0.095	142-148	0.001	175-175	0.009	354-366	0.001	289-301	0.003
3	167-179	0.001	**197-197**	**0.339**	192-232	0.001	161-182	0.007	142-154	0.001	175-208	0.001	354-372	0.001	292-292	0.024
4	167-182	0.001	197-200	0.034	195-195	0.163	161-185	0.040	142-160	0.003	175-211	0.002	354-381	0.001	292-295	0.022
5	167-185	0.001	197-209	0.011	195-198	0.004	161-188	0.065	145-145	0.019	175-214	0.001	354-384	0.001	292-298	0.002
6	170-173	0.001	197-215	0.032	195-201	0.001	161-191	0.001	145-148	0.011	178-178	0.001	354-402	0.001	292-301	0.007
7	170-176	0.001	197-218	0.013	195-207	0.003	164-182	0.001	145-151	0.004	178-181	0.002	354-421	0.001	292-304	0.003
8	173-173	0.035	197-221	0.099	195-211	0.014	167-167	0.001	145-154	0.006	178-184	0.002	354-458	0.001	**295-295**	**0.386**
9	173-176	0.004	197-224	0.022	195-214	0.002	170-170	0.001	145-160	0.025	178-208	0.001	357-357	0.022	295-298	0.063
10	173-179	0.010	197-227	0.004	195-217	0.008	170-173	0.001	145-166	0.003	**181-181**	**0.210**	357-366	0.010	295-301	0.056
11	173-182	0.020	197-236	0.003	195-220	0.050	170-185	0.001	148-148	0.127	181-184	0.024	357-372	0.001	295-304	0.046
12	173-185	0.005	200-200	0.065	195-223	0.035	173-173	0.076	148-151	0.007	181-187	0.005	357-378	0.002	295-313	0.003
13	173-193	0.004	200-203	0.003	195-226	0.057	173-182	0.001	148-154	0.012	181-190	0.007	357-384	0.003	295-317	0.003
14	173-196	0.006	200-209	0.005	195-229	0.004	173-185	0.007	148-157	0.007	181-193	0.008	357-390	0.001	295-320	0.002
15	173-199	0.002	200-215	0.005	195-232	0.008	173-188	0.016	148-160	0.088	181-196	0.007	357-396	0.005	298-298	0.023
16	176-176	0.060	200-218	0.010	195-235	0.001	173-194	0.002	148-163	0.003	181-202	0.006	357-402	0.002	298-301	0.003
17	176-179	0.006	200-221	0.025	198-198	0.027	176-176	0.001	151-151	0.043	181-205	0.015	357-408	0.006	301-301	0.192
18	176-182	0.015	200-224	0.004	198-201	0.001	176-185	0.001	151-160	0.030	181-208	0.064	357-427	0.001	301-304	0.028
19	176-185	0.001	209-209	0.033	198-204	0.001	182-182	0.030	151-163	0.003	181-211	0.079	360-366	0.001	301-317	0.008
20	176-196	0.005	209-215	0.001	198-211	0.001	182-185	0.005	151-169	0.003	181-214	0.001	360-402	0.001	301-329	0.001
21	176-199	0.002	209-218	0.001	198-220	0.002	182-188	0.003	154-154	0.035	184-184	0.041	363-421	0.001	304-304	0.034
22	176-213	0.001	209-221	0.007	198-226	0.005	185-185	0.090	154-157	0.003	184-187	0.001	**366-366**	**0.201**	304-317	0.001
23	179-179	0.080	212-212	0.002	198-238	0.001	185-188	0.025	154-160	0.037	184-190	0.001	366-372	0.010	307-317	0.003
24	179-182	0.053	215-215	0.048	201-201	0.002	185-191	0.010	154-163	0.004	184-202	0.001	366-378	0.008	313-313	0.005
25	179-185	0.004	215-218	0.003	204-204	0.005	188-188	0.158	157-157	0.032	184-205	0.002	366-381	0.002	313-317	0.007
26	179-193	0.005	215-221	0.007	204-214	0.001	188-191	0.007	157-160	0.026	184-208	0.018	366-384	0.016	317-317	0.063
27	179-196	0.022	218-218	0.027	204-217	0.001	188-194	0.003	157-172	0.003	184-211	0.009	366-390	0.002	317-320	0.002
28	179-199	0.006	218-221	0.002	204-220	0.001	191-191	0.017	**160-160**	**0.410**	184-214	0.001	366-396	0.031	320-320	0.004
29	179-210	0.001	218-224	0.001	204-226	0.001	191-194	0.002	160-163	0.033	184-217	0.001	366-402	0.024	329-329	0.004
30	179-213	0.002	221-221	0.146	207-207	0.028	194-194	0.014	160-166	0.004	187-187	0.014	366-408	0.009	332-332	0.002
31	**182-182**	**0.319**	221-224	0.015	207-211	0.003	197-197	0.001	160-169	0.002	187-196	0.001	366-415	0.001		
32	182-185	0.020	221-227	0.002	207-223	0.002			163-163	0.007	187-208	0.002	366-418	0.001		
33	182-188	0.003	224-224	0.019	207-226	0.001			166-166	0.005	187-211	0.003	366-421	0.012		
34	182-190	0.002	227-227	0.002	207-229	0.005			169-169	0.002	190-190	0.018	366-427	0.001		
35	182-193	0.010	239-239	0.004	207-232	0.002			172-172	0.002	190-193	0.001	366-433	0.001		
36	182-196	0.028	245-245	0.002	211-211	0.068					190-208	0.005	372-372	0.061		
37	182-199	0.016			211-214	0.004					190-211	0.004	372-384	0.003		
38	182-202	0.001			211-217	0.001					190-214	0.001	372-390	0.001		
39	182-205	0.001			211-220	0.012					193-193	0.010	372-396	0.007		
40	182-235	0.001			211-223	0.004					193-208	0.005	372-402	0.002		
41	185-185	0.034			211-226	0.009					193-211	0.005	372-408	0.001		
42	185-188	0.001			211-229	0.001					196-196	0.010	372-421	0.007		
43	185-190	0.001			211-232	0.002					196-199	0.002	372-427	0.004		
44	185-193	0.001			211-235	0.001					196-205	0.001	378-378	0.021		
45	185-196	0.005			214-214	0.023					196-208	0.001	378-384	0.003		

46	185-199	0.004	214-220	0.001	196-211	0.004	378-390	0.001
47	185-202	0.001	214-223	0.004	199-199	0.001	378-396	0.003
48	188-188	0.012	214-226	0.002	202-202	0.004	378-402	0.007
49	188-199	0.002	217-217	0.014	202-208	0.001	378-408	0.001
50	190-190	0.003	217-220	0.003	205-205	0.019	378-421	0.007
51	190-202	0.001	217-223	0.002	205-208	0.003	381-381	0.017
52	193-193	0.019	217-226	0.003	205-211	0.008	381-390	0.002
53	193-196	0.003	217-229	0.001	208-208	0.197	381-402	0.001
54	193-199	0.007	220-220	0.101	208-211	0.032	384-384	0.050
55	193-202	0.001	220-223	0.016	208-214	0.002	384-390	0.001
56	196-196	0.069	220-226	0.021	211-211	0.116	384-396	0.007
57	196-199	0.010	220-229	0.005	211-217	0.002	384-402	0.017
58	196-202	0.001	220-232	0.004	214-214	0.006	384-408	0.008
59	196-235	0.001	223-223	0.062	217-217	0.001	384-421	0.008
60	199-199	0.038	223-226	0.020			384-427	0.001
61	199-205	0.003	223-229	0.004			384-440	0.001
62	202-202	0.019	223-232	0.003			384-446	0.002
63	205-205	0.007	226-226	0.120			387-396	0.002
64	207-207	0.001	226-229	0.006			390-390	0.021
65	210-210	0.002	226-232	0.003			390-396	0.009
66	213-213	0.002	229-229	0.016			390-421	0.001
67	219-219	0.002	229-232	0.001			390-433	0.002
68			232-232	0.020			396-396	0.082
69			238-238	0.001			396-402	0.010
70							396-408	0.004
71							396-415	0.001
72							396-421	0.009
73							396-427	0.002
74							402-402	0.064
75							402-405	0.001
76							402-408	0.008
77							402-415	0.001
78							402-421	0.018
79							402-427	0.001
80							405-405	0.001
81							408-408	0.029
82							408-415	0.001
83							408-421	0.011
84							411-411	0.001
85							415-415	0.015
86							415-421	0.002
87							421-421	0.043
88							421-427	0.006
89							421-433	0.003
90							421-440	0.002
91							427-427	0.012
92							427-440	0.001
93							433-433	0.004
94							433-440	0.001
95							440-440	0.002
96							458-458	0.003

Supplementary Table S4. Analysis of molecular variance (AMOVA) of 1067 quinoa samples distributed among the three provinces (Chimborazo, Cotopaxi and Imbabura) and for the two collections (A and B). The analysis has been carried out considering both collections and also for each collection separately.

**Source of the variation**	**Degrees of freedom**	**Sum of squares**	**Variance component**	**% of variation**	***p*-value**
Among collections	1	77.256	0.016	0.5	0.0001
Among provinces	4	217.398	0.190	6.1	0.0001
Among samples within provinces	1061	4880.436	1.671	53.3	0.0001
Within samples/accessions	1067	1341.500	1.257	40.1	0.0001
Total	2133	6516.590			
Among provinces in Collection A	2	66.522	0.184	5.7	0.0001
Among samples within provinces	226	1164.070	2.121	66.0	0.0001
Within samples/accessions	229	208.000	0.908	28.3	0.0001
Total	457	1438.592			
Among provinces in Collection B	2	150.947	0.191	6.2	0.0001
Among samples within provinces	835	3716.295	1.549	50.1	0.0001
Within samples/accessions	838	1133.500	1.353	43.7	0.0001
Total	1675	5000.742			

Supplementary Table S5. Analysis of molecular variance (AMOVA) of the 1006 quinoa samples distributed in the two clusters (390 from Cluster 1 and 616 from Cluster 2) defined by the STRUCTURE analysis.

**Source of variation**	**Degrees of freedom**	**Sum of squares**	**Variance component**	**% of variation**	***p*-value**
Among clusters	1	297.033	0.306	9.5	0.0001
Among samples within clusters	1004	4589.365	1.659	51.6	0.0001
Within samples/accessions	1006	1260.000	1.252	38.9	0.0001
Total	2011	6146.398			

Supplementary Figure S1. Genetic structure assignment model of 1067 quinoa samples. **(a)** Evanno's method [60] with an optimal model of K = 2 genetic groups/clusters. **(b)** Grouping of Ecuadorian quinoa accession genotypes (Clusters 1 and 2). Vertical bars represent individual samples, and the color of the bar indicates the probability (membership coefficients) that a sample will be assigned to one of the identified groups (Cluster 1 in red and Cluster 2 in green).

(a)

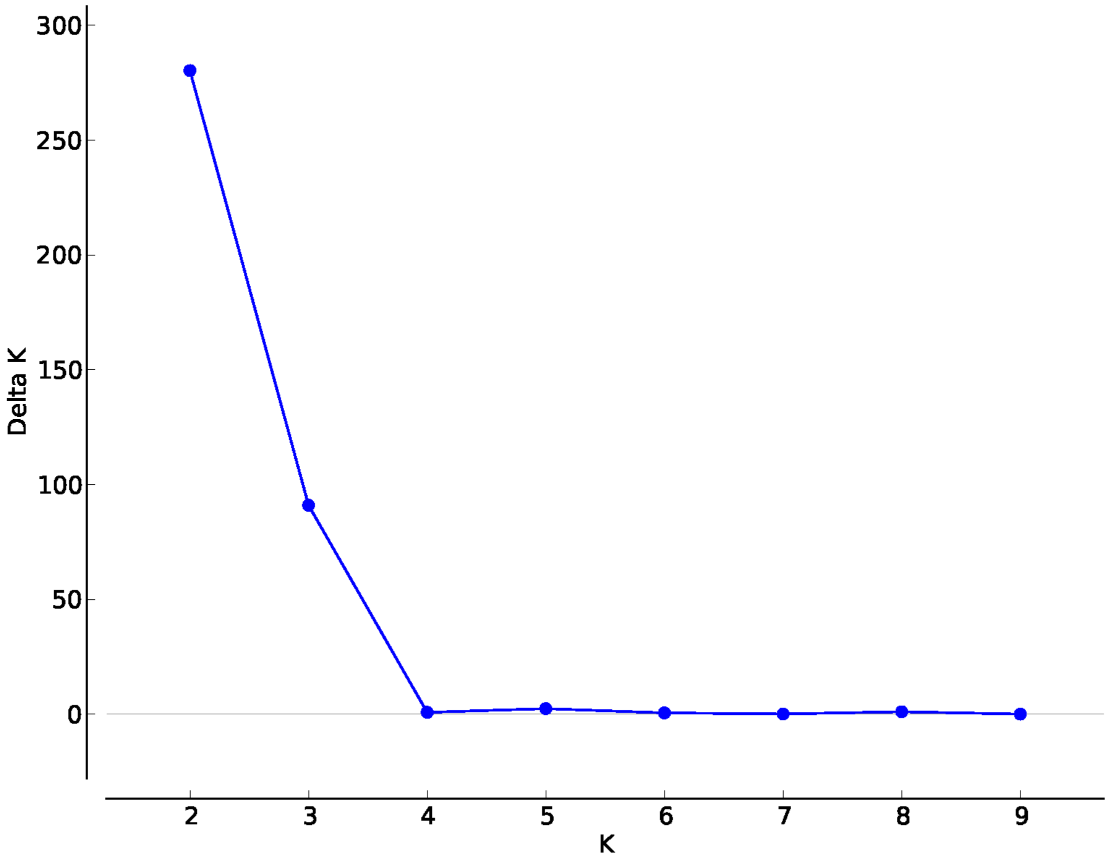

(b)

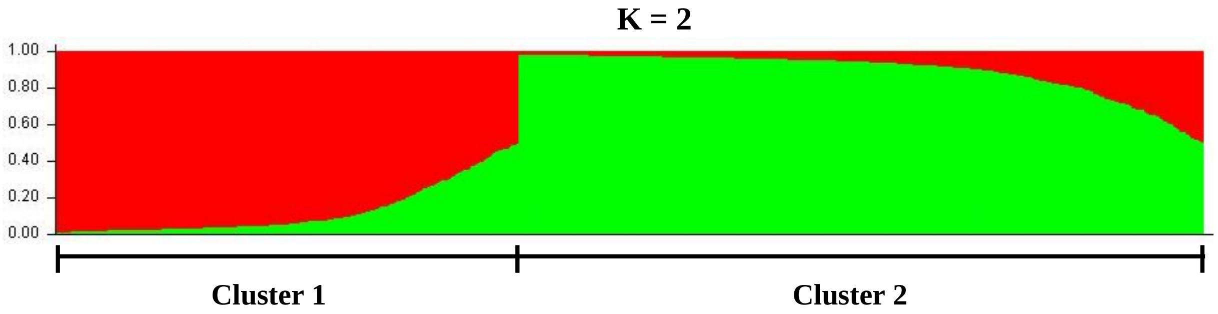

Supplement: Supplementary file 1 [file plants-14-00635-s001.zip › plants-3465014-supplementary.pdf]
